# Safety, Reactogenicity, and Health-Related Quality of Life After Trivalent Adjuvanted vs Trivalent High-Dose Inactivated Influenza Vaccines in Older Adults: A Randomized Clinical Trial

**DOI:** 10.1001/jamanetworkopen.2020.31266

**Published:** 2021-01-14

**Authors:** Kenneth E. Schmader, Christine K. Liu, Theresa Harrington, Wes Rountree, Heidi Auerbach, Emmanuel B. Walter, Elizabeth D. Barnett, Elizabeth P. Schlaudecker, Chris A. Todd, Marek Poniewierski, Mary A. Staat, Patricia Wodi, Karen R. Broder

**Affiliations:** 1 Center for the Study of Aging, Division of Geriatrics, Department of Medicine, Duke University School of Medicine, Durham, North Carolina; 2 Geriatric Research Education and Clinical Center, Durham VA Health Care System, Durham, North Carolina; 3 Section of Geriatrics, Division of Primary Care and Population Health, Stanford University, Stanford, California; 4 Geriatric Research and Education Clinical Center, Palo Alto Veterans Affairs Health Care System, Palo Alto, California; 5 Immunization Safety Office, Centers for Disease Control and Prevention, Atlanta, Georgia; 6 Duke Human Vaccine Institute, Duke University School of Medicine, Durham, North Carolina; 7 Geriatrics Section, Boston Medical Center, Department of Medicine, Boston University School of Medicine, Boston, Massachusetts; 8 Department of Pediatrics, Duke University School of Medicine, Durham, North Carolina; 9 Section of Pediatric Infectious Diseases, Boston Medical Center, Department of Pediatrics, Boston University School of Medicine, Boston, Massachusetts; 10 Cincinnati Children’s Hospital and Medical Center, Department of Pediatrics Division of Infectious Diseases, University of Cincinnati College of Medicine, Cincinnati, Ohio

## Abstract

**Importance:**

Trivalent adjuvanted inactivated influenza vaccine (aIIV3) and trivalent high-dose inactivated influenza vaccine (HD-IIV3) are US-licensed for adults aged 65 years and older. Data are needed on the comparative safety, reactogenicity, and health-related quality of life (HRQOL) effects of these vaccines.

**Objective:**

To compare safety, reactogenicity, and changes in HRQOL scores after aIIV3 vs HD-IIV3.

**Design, Setting, and Participants:**

This randomized blinded clinical trial was a multicenter US study conducted during the 2017 to 2018 and 2018 to 2019 influenza seasons. Among 778 community-dwelling adults aged at least 65 years and assessed for eligibility, 13 were ineligible and 8 withdrew before randomization. Statistical analysis was performed from August 2019 to August 2020.

**Interventions:**

Intramuscular administration of aIIV3 or HD-IIV3 after age-stratification (65-79 years; ≥80 years) and randomization.

**Main Outcomes and Measures:**

Proportions of participants with moderate-to-severe injection-site pain and 14 other solicited reactions during days 1 to 8, using a noninferiority test (5% noninferiority margin), and serious adverse events (SAE) and adverse events of clinical interest (AECI), including new-onset immune-mediated conditions, during days 1 to 43. Changes in HRQOL scores before and after vaccination (days 1, 3) were also compared between study groups.

**Results:**

A total of 757 adults were randomized, 378 to receive aIIV3 and 379 to receive HD-IIV3. Of these participants, there were 420 women (55%) and 589 White individuals (78%) with a median (range) age of 72 (65-97) years. The proportion reporting moderate-to-severe injection-site pain, limiting or preventing activity, after aIIV3 (12 participants [3.2%]) (primary outcome) was noninferior compared with HD-IIV3 (22 participants [5.8%]) (difference −2.7%; 95% CI, −5.8 to 0.4). Ten reactions met noninferiority criteria for aIIV3; 4 (moderate-to-severe injection-site tenderness, arthralgia, fatigue, malaise) did not. It was inconclusive whether these 4 reactions occurred in higher proportions of participants after aIIV3. No participant sought medical care for a vaccine reaction. No AECI was observed. Nine participants had at least SAE after aIIV3 (2.4%; 95% CI,1.1% to 4.5%); 3 had at least 1 SAE after HD-IIV3 (0.8%; 95% CI, 0.2% to 2.2%). No SAE was associated with vaccination. Changes in prevaccination and postvaccination HRQOL scores were not clinically meaningful and not different between the groups.

**Conclusions and Relevance:**

Overall safety and HRQOL findings were similar after aIIV3 and HD-IIV3, and consistent with prelicensure data. From a safety standpoint, this study’s results support using either vaccine to prevent influenza in older adults.

**Trial Registration:**

ClinicalTrials.gov Identifier: NCT03183908

## Key Points

**Question:**

What are the comparative safety, reactogenicity, and short-term effects of vaccination on health-related quality of life after trivalent adjuvanted inactivated influenza vaccine (aIIV3) or trivalent high-dose inactivated influenza vaccine (HD-IIV3) in adults aged 65 years and older?

**Findings:**

In this randomized clinical trial of 757 older adults (378 receiving aIIV3 and 379 receiving HD-IIV3), the proportion of participants with moderate-to-severe injection-site pain (primary outcome) was not higher after aIIV3 than HD-IIV3. No vaccine-related serious adverse events occurred, and postvaccination health-related quality of life was similar between aIIV3 and IIV3-HD groups.

**Meaning:**

These findings suggest that from a safety standpoint, aIIV3 or HD-IIV3 is an acceptable option to prevent influenza in older adults.

## Teaser

This randomized clinical trial assesses whether injection-site pain and reactogenicity are noninferior in trivalent adjuvanted inactivated influenza vaccine (aIIV3) compared with trivalent high-dose inactivated influenza vaccine (HD-IIV3) among adults aged 65 years and older.

## Introduction

Older adults are at high risk for severe influenza illness, cardiovascular events, hospitalization, functional decline, and death following influenza infection.^1,2,3,4,5,6,7,8,9,10^ Hospitalization rates for laboratory confirmed influenza are up to 10 times higher in people aged 65 years or older compared with younger adults.^1^ An estimated 71% to 90% of deaths from influenza occur in adults aged 65 years or older.^7,8^ The Centers for Disease Control and Prevention (CDC) Advisory Committee on Immunization Practices (ACIP) recommends annual vaccination with any US-licensed, age-appropriate, influenza vaccine.^11^ In the US, influenza vaccines that are licensed for use only in persons aged 65 years and older are trivalent high-dose inactivated influenza vaccine (HD-IIV3 [Fluzone^®^ High-Dose, licensed 2009]^12^), trivalent adjuvanted inactivated influenza vaccine (aIIV3 [Fluad^®^, licensed 2015]^13^), quadrivalent high-dose inactivated influenza vaccine (HD-IIV4 [Fluzone^®^ High-Dose Quadrivalent, licensed 2019]^14^), and quadrivalent inactivated adjuvanted influenza vaccine (aIIV4 [Fluad^®^ Quadrivalent, licensed 2020]^15^).

During the 2020 to 2021 influenza season, it is anticipated that HD-IIV4 will replace HD-IIV3.^11,16^ Both aIIV4 and aIIV3 products are expected to be distributed during the 2020 to 2021 influenza season.^11^ In addition to these age-specific vaccines, quadrivalent standard-dose, unadjuvanted inactivated influenza vaccines (SD-IIV4) and quadrivalent recombinant influenza vaccine (RIV4) are licensed and recommended for people aged 65 years and older.^11^

Studies have observed modestly higher effectiveness of HD-IIV3 and aIIV3 compared with trivalent standard-dose, unadjuvanted inactivated influenza vaccines (SD-IIV3).^17,18^ A study of Medicare beneficiaries aged 65 years and older observed that nearly three-quarters of vaccinees received aIIV3 or HD-IIV3 during the 2017 to 2018 influenza season.^19^ Safety and reactogenicity of these vaccines may also be a factor in the choice of vaccine. aIIV3 and HD-IIV3 have higher proportions of some local and systemic reactions compared with SD-IIV3.^20,21^ Safety of aIIV3 and HD-IIV3 has not been compared directly in the same clinical trial in the US, nor has the relative impact of aIIV3 and HD-IIV3 reactions on short-term, postvaccination health-related quality of life (HRQOL) in older adults.

We performed a clinical trial to determine comparative safety, reactogenicity, and short-term effects of vaccination on HRQOL after aIIV3 or HD-IIV3 in adults aged 65 years or older. The primary objective (statistical end point) was to compare proportions of participants with moderate-to-severe injection-site pain following aIIV3 vs HD-IIV3. A coprimary objective was to compare serious adverse events (SAEs) and adverse events of clinical interest (AECI) after each vaccine. Secondary objectives were to compare proportions of local and systemic reactions and changes in HRQOL after aIIV3 and HD-IIV3 vaccination.

## Methods

### Study Design and Participants

We conducted a prospective, randomized, blinded clinical trial at CDC-sponsored Clinical Immunization Safety Assessment (CISA) Project^22^ centers during the 2017 to 2018 (Duke University Medical Center and Boston Medical Center) and 2018 to 2019 (Duke, Boston, and Cincinnati Children’s Hospital Medical Center) influenza seasons. The study protocol was approved by institutional review boards at each study site; CDC relied on the Duke institutional review board. Participants provided written informed consent. The trial protocol and statistical analysis plan are provided in Supplement 1. This study followed the Consolidated Standards of Reporting Trials (CONSORT) reporting guideline.

Eligibility criteria included age greater than or equal to 65 years, living in the community, no immunosuppression as a result of an underlying illness or treatment, no use of anticancer chemotherapy or radiation therapy within the preceding 12 months, without dementia, able to speak English, and no contraindications to influenza vaccination (all criteria in eAppendix 1 in Supplement 2). We aimed to have at least 20% of enrolled participants be aged 80 years or older.

After obtaining written informed consent on day 1, study staff screened potential participants for cognitive impairment with the Mini-Cog test.^23^ Staff administered the Rowland Universal Dementia Assessment Scale (RUDAS) to adults whose Mini-Cog score was 2, which is considered a borderline score for dementia.^23,24^ Persons who scored 23 or higher (range, 0-30) on the RUDAS were deemed eligible. Staff collected demographic, medical history, medication, and influenza immunization information on each participant. Race/ethnicity was defined by the participants. Participants were randomized (1:1) to receive aIIV3 or HD-IIV3 using a permuted block randomization scheme stratified by study site. Separate permuted blocks were used for participants aged 65 to 79 years and at least 80 years. Participants and study staff performing data collection and analysis were blinded to treatment allocation. Because there was a visual difference between aIIV3 and HD-IIV3, staff who prepared and administered study vaccines were unblinded but did not participate in data collection, outcome measurement, or analysis.

Following randomization, a 0.5 mL intramuscular dose of either egg-based, US-licensed aIIV3 or HD-IIV3 was administered in the deltoid muscle. Each aIIV3 dose contained 15 µg of hemagglutinin (HA) from each of the 3 recommended influenza strains for the respective season and MF59 adjuvant, a squalene-based, oil-in-water emulsion. Each HD-IIV3 dose contained 60 µg of HA from each of the 3 recommended influenza strains for the respective season.

### Safety and Reactogenicity Assessments

Study staff monitored participants in the clinic for at least 15 minutes postvaccination for adverse events, including anaphylaxis and syncope, and assessed solicited reactogenicity events and unsolicited adverse events after vaccination on day 1 (vaccination day) through day 8 using a standard symptom diary. Local reactions assessed included injection-site pain, tenderness, swelling, redness, and shoulder pain on the vaccination side. Systemic reactions assessed were fatigue, malaise, myalgia, headache, arthralgia, nausea, vomiting, diarrhea, fever, and chills. Participants received a study thermometer, ruler, and education about completing the diary. Participants then self-graded the severity of their reactions. Reaction severity was based on criteria used in the prelicensure trials of aIIV3 and HD-IIV3: none (grade 0) mild (grade 1), moderate (grade 2), or severe (grade 3)^12,13^ (see eAppendix 2 in Supplement 2). Study staff contacted participants on day 3 and day 9 postvaccination to review solicited reactogenicity data, and assess for unsolicited adverse events, SAEs, AECI, and any new medical conditions or change in medications. Staff also monitored study participants for these outcomes, except for solicited reactogenicity, through day 43. SAEs were defined in accordance with the US Food and Drug Administration.^25^ AECIs were syncope during postvaccination monitoring in clinic, anaphylaxis within 24 hours after vaccination, and new-onset, immune-mediated conditions (including Guillain-Barré Syndrome) through day 43. Study investigators assessed relatedness of SAEs or AECI to the study vaccines.

### Health-Related Quality of Life Assessment

Study staff assessed HRQOL prevaccination on day 1 (in clinic) and on day 3 (by phone or in person) using the EuroQol 5 Dimensions-5 Level (EQ-5D-5L) and EQ Visual Analogue Scale (EQ-VAS) (2017-2018 and 2018-2019 influenza seasons) and the Late-Life Function & Disability Instrument–Computer Adaptive Test (LLFDI-CAT) (2017-2018 influenza season only).^26,27,28^ The EQ-5D-5L is a standardized, generic measure of health status that provides information about HRQOL and activities of daily living relevant to older adults: mobility, self-care, usual activities, pain or discomfort, and anxiety or depression.^26,27^ The EQ-VAS is a visual analogue scale from 0 to 100 that measures the respondent’s self-rated health.^26^ The LLFDI-CAT is a questionnaire assessing function (ability to perform discrete actions or activities as part of daily routines) and disability (socially-defined tasks) in community-dwelling older adults.^28^

### Outcome Measures

The primary outcome was comparison of proportions of participants with moderate-to-severe injection-site pain during days 1 to 8. We selected pain as the primary outcome because injection-site pain would be causally related to the vaccine, it was reported for both vaccines in prelicensure trials,^12,13^ and pain that leads to a limitation (moderate) or complete inability (severe) to perform normal daily activities is clinically meaningful. We hypothesized that the proportion of participants with moderate-to-severe pain after aIIV3 would be noninferior to that after HD-IIV3. aIIV3 was the first adjuvanted influenza vaccine used in the US.^13^ HD-IIV3 was selected as the comparator because at the time of our study, it was widely used, with substantive evidence supporting its safety.^29,30^ The coprimary outcome was comparison of the frequencies of SAEs and AECI in the 2 treatment groups. Secondary outcomes included a comparison of postvaccination moderate-to-severe local and systemic reactions during days 1 to 8, and before (day 1) vs after vaccination (day 3) changes in EQ-5D-5L utility index, EQ-VAS, and LLFDI-CAT scores. We also assessed the above outcomes after aIIV3 and HD-IIV3 by age group (aged 65-79 years and ≥80 years).

### Statistical Analysis

The planned sample size of at least 668 evaluable participants (334 per group across all sites) provided at least 80% power to reject the null hypothesis that the proportion of participants with moderate-to-severe injection-site pain after aIIV3 is inferior to HD-IIV3 during days 1 to 8 (US Food and Drug Administration package inserts). Anticipating dropout, the study aimed to enroll at least 720 participants. The statistical testing for the primary outcome was conducted at the 1-sided α = .025 level using the upper bound of a stratified by site Newcombe binomial confidence interval^31^ with Cochran-Mantel-Haenszel (CMH) weighting with a noninferiority margin of 5%. The statistical method above was also used for the secondary comparison of the difference in proportions of moderate-to-severe local and systemic reactions between allV3 and HD-IIV3 groups, with a 1-sided α = 0.01 significance level to adjust for multiple comparisons. The reactogenicity comparisons were made using the full analysis population 1 that consisted of all participants who were randomized, vaccinated, and provided at least 1 day of complete data on the symptom diary form. The comparison of the frequencies of SAEs and AECI in the 2 treatment groups was made using exact binomial 95% CIs. The comparisons of at least 1 severe local and/or systemic reaction were made using the full analysis population 1 with a Fisher exact test. The changes in HRQOL after allV3 and HD-IIV3 administration were evaluated using Mann-Whitney *U* tests. These comparisons were made using the full analysis population 2 that consisted of all participants who were randomized and vaccinated. For the HRQOL comparisons, we used a 2-sided α = .01 level in the study population and α = .05 level for age-group analyses, which were considered exploratory. These data were analyzed using SASstatistical software version 9.4 (SAS Institute). Statistical analysis was performed from August 2019 to August 2020.

## Results

### Study Participants

**Figure 1.**
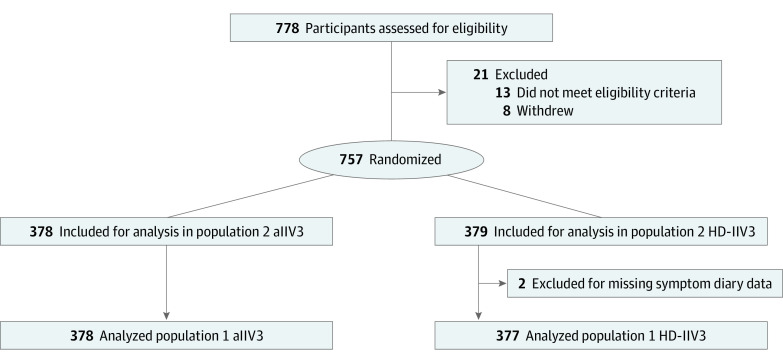
Study Enrollment Flowchart allV3 indicates trivalent adjuvanted inactivated influenza vaccine; and HD-IIV3, trivalent high-dose inactivated influenza vaccine.

We assessed 778 participants for eligibility during 2 influenza seasons. Eight participants withdrew, and 13 additional participants did not meet eligibility criteria, yielding 757 randomized participants; 378 received aIIV3 and 379 received HD-IIV3 in full analysis population 2 (Figure 1). Of these 757 randomized participants, there were 420 women (55%) and 589 White individuals (78%) with a median (range) age of 72 (65-97) years. The baseline demographic and clinical characteristics of randomized participants were similar between the 2 study groups (Table 1).

**Table 1.** Baseline Demographic and Clinical Characteristics of Participants in the Trivalent Adjuvanted Inactivated Influenza Vaccine and Trivalent High-Dose Inactivated Influenza Vaccine Groups

Characteristic	Patients, No. (%)	
	aIIV3 (n = 378)	HD-IIV3 (n = 379)
Study site		
Duke	214 (56.6)	214 (56.5)
Boston	121 (32.0)	122 (32.2)
Cincinnati	43 (11.4)	43 (11.3)
Influenza season of enrollment		
2017-2018	138 (49.5)	141 (50.5)
2018-2019	240 (50.2)	238 (49.8)
Age, median (range), y	72 (65-96)	72 (65-97)
Age group, y		
65-79	298 (78.8)	296 (78.1)
≥80	80 (21.2)	83 (21.9)
Female	213 (56.3)	207 (54.6)
Race		
White	286 (75.7)	303 (79.9)
Black	70 (18.5)	59 (15.6)
Asian	5 (1.3)	4 (1.1)
Other^a^	17 (4.5)	13 (3.4)
Hispanic ethnicity	7 (1.9)	1 (0.3)
Education, some college or higher	328 (86.7)	331 (87.3)
Employment		
Employed	62 (16.4)	64 (16.9)
Retired	300 (79.4)	300 (79.2)
None	15 (4)	14 (3.7)
Living alone	151 (39.9)	148 (39.1)
Cardiovascular and respiratory disorders^b^		
Atrial fibrillation	29 (7.7)	22 (5.8)
Coronary artery disease	28 (7.4)	27 (7.1)
Heart failure	23 (6.1)	13 (3.4)
Hyperlipidemia	137 (36.2)	151 (39.8)
Hypertension	83 (22.0)	71 (18.7)
Valvular heart disease	17 (4.5)	11 (2.9)
Asthma	7 (1.9)	8 (2.1)
Chronic obstructive pulmonary disease	6 (1.6)	1 (0.3)
Other common conditions		
Arthritis	73 (19.3)	75 (19.8)
Depression	50 (13.2)	49 (12.9)
Diabetes	23 (6.1)	27 (7.1)
Gastroesophageal reflux disease	39 (10.3)	23 (6.1)
Hearing loss	8 (2.1)	16 (4.2)
Hypothyroidism	38 (10.1)	34 (9.0)
Statin use	184 (48.7)	177 (46.7)
Received influenza vaccine in the previous season^c^	276 (73.1)	279 (73.6)
Prevaccination, mean (SD)		
EuroQol 5 Dimensions-5 Level^d^	0.89 (0.118)	0.90 (0.130)
EuroQol Visual Analogue Scale^e^	85.5 (13.672)	85.8 (12.506)

Abbreviations: aIIV3, trivalent adjuvanted inactivated influenza vaccine; HD-IIV3, trivalent high-dose inactivated influenza.

^a^ Other races include American Indian/Alaskan Native, Asian, and more than 1 race.

^b^ These conditions are not mutually exclusive.

^c^ Determined by medical records or self-report.

^d^ The score is a utility index that ranges from −0.109 (worst health) to 1.000 (best health) for US-specific values.

^e^ EuroQol Visual Analogue Scale measures self-rated health with a score that ranges from 0 to 100, with 100 equal to the “best health you can imagine” and 0 equal “to the worst health you can imagine.”

We randomized 279 participants in 2017 to 2018 and 478 participants in 2018 to 2019. No study participant received study vaccines more than once in successive years. For full analysis population 1, 378 received aIIV3 and 377 received HD-IIV3 because 2 participants were missing symptom diary data (Figure 1).

### Safety and Reactogenicity

**Figure 2.**
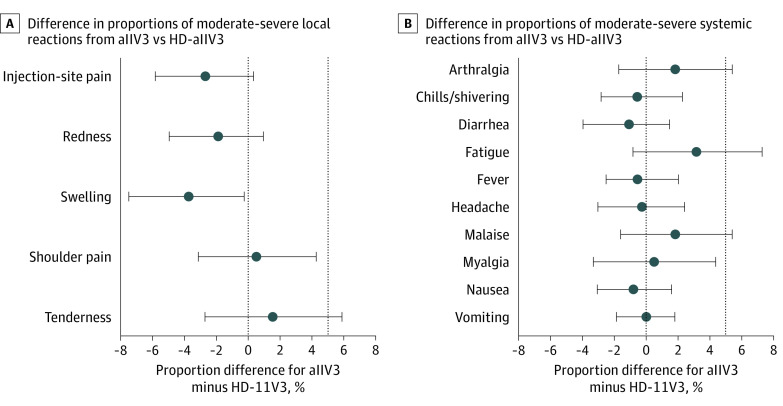
Difference in Trivalent Adjuvanted Inactivated Influenza Vaccine (aIIV3) vs Trivalent High-Dose Inactivated Influenza Vaccine (HD-IIV3) Reactions Circles denote means and error bars denote 98% CIs except for injection-site pain, for which a 95% CI is shown. Dashed lines at 0% denote no difference; dashed lines at 5% denote the noninferiority margin.

The proportion of participants reporting moderate-to-severe injection-site pain after aIIV3 (12 patients [3.2%]) was noninferior to (ie, not higher than) the proportion of patients reporting pain after receiving HD-IIV3 (22 patients [5.8%]) (difference, −2.7%; 95% CI, −5.8% to 0.4%) (Table 2 and Figure 2). The difference of proportions (aIIV3 minus HD-IIV3) of moderate-to-severe reactions for 4 solicited symptoms (injection-site tenderness, arthralgia, fatigue, and malaise) did not meet noninferiority criteria for aIIV3 (Table 2 5.8%Figure 2). These findings are inconclusive because the confidence intervals include the noninferiority margin and do not exclude 0.^32^ aIIV3 was noninferior to HD-IIV3 for the remaining 10 moderate-to-severe solicited reactions (Table 2 5.8%Figure 2). We observed clinically similar patterns of moderate-to-severe systemic reactions in the full study population (Table 2) and in the age groups for both 65 to 79 years and at least 80 years (eTable in Supplement 2). In the 163 participants aged 80 years or older, the proportion of participants with moderate-to-severe reactions were low and no clinically meaningful differences were observed between the 2 vaccine groups.

**Table 2.** Local and Systemic Reactions During Days 1 Through 8 Following Trivalent Adjuvanted Inactivated Influenza Vaccine and Trivalent High-Dose Inactivated Influenza in Older Adults

Reactions	Patients, No. (%)		Proportion difference (aIIV3 – HD-IIV3), % (95% CI)^a^
	aIIV3 (n = 378)	HD-IIV3 (n = 377)	
Local			
Injection-site pain			
Any	81 (21.4)	95 (25.2)	−2.7 (−5.8 to 0.4)^d^
Moderate^b^	10 (2.6)	21 (5.6)	
Severe^c^	2 (0.5)	1 (0.3)	
Moderate-to-severe	12 (3.2)	22 (5.8)	
Tenderness			
Any	177 (46.8)	181 (48.0)	1.6 (−2.7 to 5.9)^e^
Moderate^b^	24 (6.3)	20 (5.3)	
Severe^c^	3 (0.8)	1 (0.3)	
Moderate-to-severe	27 (7.1)	21 (5.6)	
Swelling			
Any	43 (11.4)	49 (13.0)	−3.7 (−7.5 to −0.25)^d^
Moderate^b^	6 (1.6)	20 (5.3)	
Severe^c^	3 (0.8)	3 (0.8)	
Moderate-to-severe	9 (2.4)	23 (6.1)	
Redness			
Any	30 (7.9)	36 (9.5)	−1.9 (−5.0 to 1.0)^d^
Moderate^b^	4 (1.1)	9 (2.4)	
Severe^c^	2 (0.5)	4 (1.1)	
Moderate-to-severe	6 (1.6)	13 (3.5)	
Shoulder pain on side of vaccination			
Any	61 (16.1)	51 (13.5)	0.5 (−3.1 to 4.3)^d^
Moderate^b^	14 (3.7)	14 (3.7)	
Severe^c^	3 (0.8)	1 (0.3)	
Moderate-to-severe	17 (4.5)	15 (4.0)	
Systemic			
Fatigue			
Any	59 (15.6)	40 (10.6)	3.2 (−0.1 to 7.3)^e^
Moderate^b^	24 (6.3)	12 (3.2)	
Severe^c^	3 (0.8)	3 (0.8)	
Moderate-to-severe	27 (7.1)	15 (4.0)	
Malaise			
Any	46 (12.2)	33 (8.8)	1.8 (−1.6 to 5.4)^e^
Moderate^b^	15 (4.0)	7 (1.9)	
Severe^c^	3 (0.8)	4 (1.1)	
Moderate-to-severe	18 (4.8)	11 (3.0)	
Myalgia			
Any	44 (11.6)	39 (10.3)	0.5 (−3.3 to 4.4)^d^
Moderate^b^	16 (4.2)	15 (4.0)	
Severe^c^	3 (0.8)	2 (0.5)	
Moderate-severe	19 (5.0)	17 (4.5)	
Headache			
Any	43 (11.4)	39 (10.3)	−0.3 (−3.0 to 2.4)^d^
Moderate^b^	4 (1.1)	6 (1.6)	
Severe^c^	3 (0.8)	2 (0.5)	
Moderate-to-severe	7 (1.9)	8 (2.1)	
Arthralgia			
Any	38 (10.1)	31 (8.2)	1.8 (−1.7 to 5.4)^e^
Moderate^b^	14 (3.7)	8 (2.1)	
Severe^c^	4 (1.1)	3 (0.8)	
Moderate-to-severe	18 (4.8)	11 (2.9)	
Nausea			
Any	21 (5.6)	15 (4.0)	−0.8 (−3.1 to 1.6)^d^
Moderate^b^	2 (0.5)	3 (0.8)	
Severe^c^	0	2 (0.5)	
Moderate-to-severe	2 (0.5)	5 (1.3)	
Diarrhea			
Any	20 (5.3)	18 (4.8)	−1.1 (−4.0 to 1.5)^d^
Moderate^b^	4 (1.1)	8 (2.1)	
Severe^c^	1 (0.3)	1 (0.3)	
Moderate-to-severe	5 (1.4)	9 (2.4)	
Vomiting			
Any	4 (1.1)	3 (0.8)	0.0 (−1.8 to 1.8)^d^
Moderate^b^	1 (0.3)	1 (0.3)	
Severe^c^	1 (0.3)	1 (0.3)	
Moderate-to-severe	2 (0.6)	2 (0.6)	
Chills			
Any	16 (4.2)	14 (3.7)	−0.5 (−2.8 to 2.3)^d^
Moderate^b^	3 (0.8)	5 (1.3)	
Severe^c^	0	0	
Moderate-to-severe	3 (0.8)	5 (1.3)	
Fever			
Any	12 (3.2)	15 (4.0)	0.5 (−2.5 to 2.0)^d^
Moderate^b^	0	3 (0.8)	
Severe^c^	1 (0.3)	0	
Moderate-to-severe	1 (0.3)	3 (0.8)	

Abbreviations: aIIV3, trivalent adjuvanted inactivated influenza vaccine; HD-IIV3, trivalent high-dose inactivated influenza.

^a^ Proportion difference of aIIV3 minus HD-IIV3 for moderate-to-severe reactions and 95% CI. Analysis of proportion difference of aIIV3 minus HD-IIV3 for moderate-to-severe reactions was the objective of the study. Analysis of differences for any, moderate alone and severe alone was not done because these were not objectives of the study.

^b^ Moderate swelling and redness was considered 25 mm to less than 50 mm; moderate fever was considered more than 38 °C but less than 39 °C; all other reactions, moderate was considered some limitation in normal daily activity.

^c^ Severe swelling and redness was considered at least 50 mm; moderate fever was considered higher than 39 °C; all other reactions, severe was considered completely unable to perform normal daily activity.

^d^ Inferiority hypothesis was rejected and noninferiority is concluded.

^e^ Inferiority hypothesis was not rejected and noninferiority cannot be concluded.

At least 1 severe local reaction occurred in 3.7% of aIIV3 recipients and 2.9% HD-IIV3 recipients. At least 1 severe systemic reaction occurred in 2.1% of aIIV3 recipients and 1.6% of HD-IIV3 recipients. At least 1 severe local or systemic reaction occurred in 4.8% of aIIV3 recipients and 4.0% HD-IIV3 recipients. No participant sought medical attention for a local or systemic reaction after vaccination on days 1 to 8.

There were no episodes of syncope during postvaccination monitoring in the clinic or anaphylaxis within 24 hours of vaccination. During the 43-day follow-up period, there were no deaths, episodes of Guillain-Barré syndrome, or new-onset immune-mediated conditions. Nine participants had at least 1 SAE after aIIV3 (2.4%; 95% CI,1.1%-4.5%); 3 participants had at least 1 SAE after HD-IIV3 (0.8%; 95% CI, 0.2%-2.2%) (Table 3). Study investigators assessed no SAE to be associated with vaccination and observed no clinical imbalances between study groups.

**Table 3.** Older Adult Participants Experiencing Serious Adverse Events During Days 1 Through 43 Following Trivalent Adjuvanted Inactivated Influenza Vaccine and Trivalent High-Dose Inactivated Influenza^a^

Group, time since vaccination, d	Sex	Age group, y	SAE description
aIIV3			
22	Male	65-79	Pulmonary emboli
23	Female	65-79	Fall
24	Male	65-79	Small bowel obstruction
27	Female	65-79	Respiratory failure^b^
27	Female	65-79	Asthma exacerbation and congestive heart failure
29	Female	65-79	Stress-induced cardiomyopathy
29	Female	65-79	Transient ischemic attack
34	Male	≥80	Fall
34	Male	65-79	Near syncope due to orthostasis
HD-IIV3			
11	Male	≥80	Chest pain
18	Female	65-79	Postoperative ileus
39	Male	≥80	Metastatic squamous cell carcinoma^b^

Abbreviations: aIIV3, trivalent adjuvanted inactivated influenza vaccine; HD-IIV3, trivalent high-dose inactivated influenza; SAE, serious adverse events.

^a^ None of the SAEs was judged to be causally related to receipt of vaccine.

^b^ Patient died from complications of SAE and underlying health comorbidities more than 43 days after vaccination.

### HRQOL Scores

Participants’ baseline prevaccination HRQOL scores were similar in both study groups (Table 1). Change in EQ-5D-5L utility index score from day 1 (before vaccination) to Day 3 postvaccination was not significantly different between treatment groups (−0.05 aIIV3 vs −0.05 HD-IIV3), nor was change in EQ VAS (−2.22 aIIV3 vs −2.49 HD-IIV3), or change in LLFDI scores for activity limitation, daily activities, basic mobility, participation restriction, social roles, and instrumental roles. Changes in EQ-5D-5L utility index score, EQ-VAS, and LLFDI scores from day 1 to day 3 postvaccination were not significantly different between treatment groups in the exploratory analyses by age group (65-79 years and ≥80 years), with the exception of the LLFDI changes in social roles scores in participants aged 65 to 79 years (−0.66 aIIV3 vs −1.98 HD-IIV3; difference, −1.32; *P* = .04).

## Discussion

To our knowledge, this is the first randomized clinical trial in the US directly comparing the safety, reactogenicity, and short-term quality of life following vaccination with aIIV3 or HD-IIV3 in older adults. Consistent with the study hypothesis, we found that the proportions of participants with moderate-to-severe injection-site pain after vaccination with aIIV3 was noninferior (not higher) vs HD-IIV3. Ten reactions met noninferiority criteria for aIIV3; 4 (moderate-to-severe injection-site tenderness, arthralgia, fatigue, and malaise) did not. It was inconclusive whether these 4 reactions occurred in higher proportions of participants.^32^ Few (4.8% of aIIV3 recipients and 4.0% HD-IIV3 recipients) individuals experienced any severe reaction after either vaccine in our study. No reactions led to a medical visit. We observed no vaccine-related SAEs or AECI, including no new-onset immune-mediated disease within 43 days after aIIV3 or HD-IIV3 vaccination.

Safety profiles of aIIV3 and HD-IIV3 in our study were consistent with those found in prelicensure studies for each vaccine. The proportions of individuals with moderate-to-severe injection-site pain were 4.2% of aIIV3 recipients and 4.0% of HD-IIV3 recipients in prelicensure studies,^12,13^ compared with 3.2% and 5.8%, respectively, in our study. Moderate-to-severe injection-site tenderness after aIIV3 was 4% higher in our study than in prelicensure aIIV3 studies^13^; injection-site swelling after HD-IIV3 was 3% higher in our study than in prelicensure studies.^12^ Prelicensure studies compared reactogenicity and safety of aIIV3 and HD-IIV3 to SD-IIV3.^12,13^

In a postlicensure study in Hong Kong, Cowling et al^33^ assessed reactogenicity and safety outcomes in older adults receiving aIIV3 (508 patients) or HD-IIV3 (510 patients). Similar to our study, the most commonly reported reactions were injection-site pain and tenderness. Proportions of any and moderate-to-severe reactions at 1 to 2 days after aIIV3 or HD-IIV3 were generally lower than in our study. Hospitalization rates less than or equal to 30 days after vaccination were similar between aIIV3 (0.6%) and HD-IIV3 (1%). Investigators identified no vaccine-related SAEs.^33^

In a recently published study from Australia,^34^ the AusVaxSafety postlicensure active surveillance program analyzed text responses from more than 47 000 persons aged at least 65 years who received aIIV3 or HD-IIV3. Injection-site pain was the most frequently reported solicited adverse event (2.1% for HD-IIV3 and 1.3% for aIIV3). Rates of medical attention (used as a proxy for serious AEs) were low for both HD-IIV3 and aIIV3 (0.3%). Similar to our study, results of this comparative safety study of aIIV3 and HD-IIV3 in older adults were reassuring.

For older persons, any reaction that limits or prevents activities of daily living may have a substantial impact on quality of life. In our study, we measured quality of life change from baseline to 2 days postvaccination using HRQOL instruments validated for older adults. We found no significant differences in the change of prevaccination vs postvaccination HRQOL scores between the aIIV3 and HD-IIV3 groups. Changes in scores from prevaccination to 2 days postvaccination in each group were small, and clinically not meaningful. To our knowledge, our study is the first clinical trial to assess postvaccination HRQOL effects in adults receiving aIIV3 or HD-IIV3 vaccines.

### Limitations

This study has several limitations. First, our study was powered for a single outcome of injection-site pain, but the study was not powered for other reactions or study outcomes. In addition, the study population was drawn from a population of community-dwelling older adults, who were highly educated and had high self-rated health. Postvaccination HRQOL and VAS within group and between groups might have been different if study participants resided in assisted living facilities or had lower baseline HRQOL and VAS scores. Also, our study does not address safety of administering aIIV3 or HD-IIV3 with other vaccines at the same visit.

The safety of adjuvanted influenza vaccines has been a topic of public interest.^35^ We found that our primary outcome of moderate-to-severe injection-site pain after aIIV3 was not worse than HD-IIV3. Of the 14 other reactions assessed as secondary outcomes, 10 (including fever), were not higher after aIIV3 than HD-IIV3. Our study findings are inconclusive as to whether the proportion of participants with injection-site tenderness and 3 other reactions were significantly higher after aIIV3, compared with HD-IIV3, suggesting that our study was underpowered for these secondary reactogenicity outcomes.^32^ The findings that no reaction led to a medical visit in either group and that there was not an important impact on short-term quality of life after vaccination lends further support to the safety of both vaccines. Similar clinical safety profiles support the conclusion that concerns about differences in safety between aIIV3 and HD-IIV3 need not be a factor when choosing between them.

## Conclusions

In our trial comparing aIIV3 and HD-IIV3 in older adults, no vaccine-related SAEs occurred and safety findings were consistent with prelicensure data. The proportion of participants with moderate-to-severe injection-site pain was not higher after aIIV3 than HD-IIV3. Postvaccination HRQOL was similar. From the standpoint of safety, our study’s results suggest that either vaccine is an acceptable option to prevent influenza in older adults.

## Notes

Supplement 1. **Trial Protocol and Statistical Analysis Plan**

Supplement 2. **eAppendix 1.** Eligibility Criteria **eAppendix 2.** Reactogenicity Assessment **eTable.** Local and Systemic Reactions during Days 1 through 8 Following Trivalent Adjuvanted Inactivated Influenza Vaccine (aIIV3) and Trivalent High-Dose Inactivated Influenza Vaccine (HD-IIV3) by Age Group in Older Adults

Supplement 3. **Data Sharing Statement**
