# Supplementary material for: Safety, Reactogenicity, and Health-Related Quality of Life After Trivalent Adjuvanted vs Trivalent High-Dose Inactivated Influenza Vaccines in Older Adults: A Randomized Clinical Trial
Source: JAMA Netw Open. 2021 Jan 14;4(1):e2031266. doi: 10.1001/jamanetworkopen.2020.31266 (PMC7809592; doi:10.1001/jamanetworkopen.2020.31266)
Supplement: Supplement 1. — Trial Protocol and Statistical Analysis Plan [file jamanetwopen-e2031266-s001.pdf]

Safety and Immunogenicity of Adjuvanted versus High-Dose Inactivated Influenza Vaccines in Older Adults

Short Title: FLUAD® vs. Fluzone® High-Dose Study

Centers for Disease Control & Prevention
Clinical Immunization Safety Assessment (CISA) Project

Lead Site (Duke University) Principal Investigator: Ken Schmader, MD
Lead Site Sub-principal Investigator: Emmanuel B. Walter, MD, MPH

Contributing Site (Boston University) Principal Investigator: Elizabeth Barnett, MD
Contributing Site Sub-principal Investigator: Christine Liu, MD
Contributing Site Sub-principal Investigator: Heidi Auerbach, MD

Contributing Site (Cincinnati Children's Hospital Medical Center) Principal Investigator: Elizabeth Schlaudecker, MD
Contributing Site Sub-principal Investigator: Mary Staat, MD

Centers for Disease Control and Prevention (CDC) Principal Investigator: Theresa Harrington, MD, MPH&TM
CDC Sub-principal Investigator: Karen R. Broder, MD

NCT # NCT03183908

Version Number: 5.0

25 September 2018

STATEMENT OF COMPLIANCE

- This trial will be conducted in compliance with the protocol, the International Conference on Harmonization (ICH) Guideline E6—Good Clinical Practice (GCP), and the applicable guidelines and regulatory requirements from the United States (US) Code of Federal Regulations (CFR), 45 CFR Part 46.
- All study personnel with subject contact have completed Human Subjects Protection Training.

TABLE OF CONTENTS

PROTOCOL SUMMARY 5
1 BACKGROUND 10
1.1 Background 10
1.2 Summary and Rationale 11
2 STUDY OBJECTIVES AND OUTCOME MEASURES 11
2.1 Study Objectives 11
2.1.1 Primary Objectives: 11
2.1.2 Secondary Objectives 12
2.1.3 Exploratory Objectives: 12
2.2 Study Outcome Measures 12
2.2.1 Primary Outcome Measures: 12
2.2.2 Secondary Outcome Measures 13
2.2.3 Exploratory Outcome Measures 13
3 STUDY DESIGN 14
3.1 Main study design 14
3.2 Laboratory Studies 14
4 STUDY ENROLLMENT AND WITHDRAWAL 15
4.1 Subject Inclusion Criteria 15
4.2 Subject Exclusion Criteria 15
4.3 Recruitment 16
4.4 Reasons for and Handling of Withdrawals 18
4.5 Termination of Study 18
5 STUDY SCHEDULE, PROCEDURES, & EVLAUTIONS 18
5.1 Schedule of events and data collection 18
5.2.1 Randomization 22
5.3 Data Collection 23
5.3.1 Vaccine Supply, Storage, Administration, and Blinding 23
5.4 Reactogenicity and Safety Assessment 24
5.4.2 Reporting of Adverse Events 27
5.4.3 Safety Monitoring Plan 28
5.5 Health-Related Quality of Life (HRQOL) 28
5.5.1 Generic Measures of HRQOL 28
5.5.2 Vaccination Specific Measure of HRQOL 30
5.5.3 Perceptions of the Vaccination Experience and Methods of Adverse Event Monitoring 30
5.6 Health Care Utilization 30
5.7 Biospecimens Collection & Handling 30
5.7.1 Serum 30
6 LABORATORY ANALYSES 31

6.1 Influenza Hemagglutination Inhibition (HAI) Assay 31
7 STATISTICAL CONSIDERATIONS 31
7.1 Analysis Plan 32
7.1.1 Sample Size 32
7.1.2 Analysis Populations 32
7.1.3 Primary Objective 1 33
7.1.4 Primary Objective 2 33
7.1.5 Primary Objective 3 33
7.1.6 Secondary Objective 1 34
7.1.7 Secondary Objective 2 34
7.1.8 Secondary Objective 3 35
7.1.9 Sensitivity Analyses 35
7.1.10 Exploratory Objectives 36
7.2 Data Management 36
7.2.1 Research Electronic Data Capture (REDCap) 36
7.3 Role of the CDC Investigators in the Project 37
8 HUMAN SUBJECTS 37
8.1 Human Subjects Involvement, Characteristics, and Design 37
8.2 Sources of Material 38
8.3 Potential Risks and Benefits 38
8.4 Adequacy of Protection Against Risks 39
8.4.1 Protections against Risk 39
8.4.2 ClinicalTrials.gov Requirements 39
8.5 Human Subjects 39
8.5.1 Vulnerable Subjects Research 39
REFERENCES 41
APPENDIX 43
Appendix A: Written Informed Consent 44
Appendix B: Mini-Cog 56
Appendix C: Rowland Universal Dementia Assessment Scale (RUDAS) 59
Appendix D: Treatment Administration Record/Immediate Reactogenicity Assessment Form 62
Appendix E: Patient Symptom Diary 65
Appendix F: FACES Pain Scale 73
Appendix G: Immune-Mediated Conditions 75
Appendix H: Safety Monitoring Plan 77
Appendix I: LLFDI-CAT 80
Appendix J: EQ-5D-5L and VAS 118
Appendix K: Vaccine Reaction Questionnaire 121
Appendix L: Perceptions of the Vaccination Experience 125
Appendix M: Recruitment Letter 132
Appendix N: Flyer 133
Appendix O: Phone Script 135

PROTOCOL SUMMARY

**Title:**	Safety and Immunogenicity of Adjuvanted versus High-Dose Inactivated Influenza Vaccines in Older Adults
**Phase:**	Phase IV
**Population:**	≥880 community-dwelling adults ≥65 years of age who intend to receive the inactivated influenza vaccine (IIV) during the 2017-2018 (Year 1) or 2018-2019 (Year 2) influenza season. Approximately 20% of the participants will be ≥80 years old.
**Clinical Sites:**	Three: Duke University (Lead); Boston Medical Center (Contributing); Cincinnati Children's Hospital (Contributing Subcontractor)
**Study Duration:**	24 months total<br>• 18 months to recruit/enroll over two influenza seasons<br>• 6 weeks participation for most subjects<br>• Maximum of 6 months participation for a subset of approximately 100 participants at Duke during 2017-2018 flu season<br>• 4.5 months to perform analysis and laboratory assays after the end of each flu season<br>• A subset of 60 subjects from year 1 will be revaccinated with same influenza vaccine product during the year 2 influenza season at Duke
**Participant Duration:**	• 43 days for 780 participants<br>• 181 days for approximately 100 participants at Duke for longer term immunogenicity studies (Year 1 only)<br>• Approximately 14 months for 60 participants in the repeat vaccination sub-study (at Duke only)
**Description of Study Procedures:**	This is a prospective, randomized, blinded clinical trial to assess the safety and immunogenicity of adjuvanted inactivated influenza vaccine (aIIV3) versus High-Dose inactivated influenza vaccine (IIV3-HD) in subjects aged ≥65 years. Participants aged 65 to 79 years will be randomized (1:1) to receive either aIIV3 or IIV3-HD using a permuted block randomization scheme stratified by Lead and Contributing Site(s) for at least 704 subjects. A separate permuted block will be allocated for at least 176 subjects who are age 80 years or older, thus achieving an overall N≥880.

	Vaccine reactogenicity will be assessed for 8 days post-injection and compared between the two groups using identical paper diaries. Serious adverse events and events of clinical interest will be assessed through 42 days post-vaccination and compared between the two groups.<br><br>Health-related quality of life will be assessed at baseline before vaccination, at day 3 and day 9 post-vaccination. Perceptions of the vaccination study experience and adverse event monitoring experience will be assessed at days 9 and 29 post-vaccination.<br><br>Vaccine strain-specific serum hemagglutination inhibition antibody titers will be measured from blood samples collected pre- and 29 days post-immunization for all participants and for approximately 100 subjects at Duke on Day 181 (Year 1 only).<br><br>Vaccine reactogenicity, serious adverse events (SAEs) and events of clinical interest, health related quality of life, and vaccine strain-specific serum hemagglutination inhibition antibody titers pre- and 29 days post-immunization will be measured in a subset of 60 subjects in a repeat vaccination sub-study (at Duke only). Subjects in the second year of the repeat vaccination sub-study will be assigned to receive the same vaccination that they received the first year.
**Objectives:**	Primary Objectives:<br>1. To compare the proportions of moderate/severe injection-site pain after aIIV3 and IIV3-HD in the full study population<br>a. Hypothesis: the proportion of subjects who have moderate/severe injection site pain within the first week post-vaccination will be non-inferior for allV3 compared to IIV3-HD in the full study population<br>2. To compare serious adverse events and events of clinical interest after aIIV3 and IIV3-HD in the full study population and by age-group<br>3. To compare the seroconversion rate for the H3N2 influenza A strain after aIIV3 and IIV3-HD in the full study population

	a. Hypothesis: the seroconversion rate for the H3N2 influenza A strain in the full study population after aIIV3 will be non-inferior to IIV-HD<br><br>Secondary Objectives<br>1. To compare the proportions of local and systemic reactions (other than moderate/severe injection site pain) after aIIV3 and IIV3-HD in the full study population and by age-group (65-79 years and ≥80 years)<br>2. To describe and compare changes in health-related quality of life after aIIV3 and IIV3-HD in the full study population and by age-group<br>3. To compare serum hemagglutination inhibition (HAI) antibody titers after aIIV3 and IIV3-HD for each of the three influenza vaccine strains contained in the respective vaccine for that season in the full study population and by age (except for seroconversion for the H3N2 strain in the full study population)<br><br>Exploratory Objectives:<br>1. To describe how the reactogenicity events affect health-related quality of life after IIV3 and IIV3-HD in the full study population and by age-group<br>2. To describe and compare participant perceptions of the vaccination experience<br>3. To describe participant perceptions of methods of adverse event monitoring in older adults receiving influenza vaccines.<br>4. To describe injection site pain immediately after vaccination after aIIV3 and IIV3-HD<br>5. To describe and compare the proportions of immediate adverse events after aIIV3 and IIV3-HD and unsolicited adverse events in the full study population and by age-group (65-79 years and ≥80 years)<br>6. To describe factors (e.g., statin use) associated with reactogenicity to aIIV3 and IIV3-HD<br>7. To describe the safety of aIIV3 and IIV3-HD after repeat administration of the same product over 2 consecutive influenza seasons in a subset of subjects<br>8. To describe the relationship between reactogenicity and immunogenicity for aIIV3 and IIV3-HD<br>9. To explore factors (e.g., statin use) associated with immunogenicity for aIIV3 and IIV3-HD<br>10. To describe the immunogenicity of aIIV3 and IIV3-HD

	after repeat administration of the same product over 2 consecutive influenza seasons in a subset of subjects<br>11. To assess changes in serum hemagglutination inhibition at 1 month after aIIV3 and IIV3-HD vaccination and at 6 months after vaccination in a subset of subjects
**Outcome Measures:**	Primary:<br>1. Comparison of the proportion of subjects reporting moderate/severe injection site pain within the first week post-vaccination in both treatment groups.<br>2. The frequency and descriptions of serious adverse events and adverse events of clinical interest observed in the two treatment cohorts<br>3. H3N2 HAI seroconversion: The proportion of subjects achieving H3N2 seroconversion at day 29 (an HAI titer ≥ 1:40 at day 29 if the baseline titer is < 1:10 or a minimum four-fold rise in HAI titer if the baseline titer is ≥ 1:10) in the respective season's vaccine<br><br>Secondary<br>1. Comparison of local and systemic reactions within the first week post-vaccination in both treatment groups.<br>2. Change in scores on the Late Life Function & Disability Instrument (Year 1 only), EuroQOL 5 dimensions-5 level (EQ-5D-5L) and EuroQOL visual analogue scale (EQ VAS) pre-vaccination and post-vaccination will be compared between the vaccination groups and age groups.<br>3. HAI titers:<br>a. The proportion of subjects achieving seroconversion at day 29 (an HAI titer ≥ 1:40 at day 29 if the baseline titer is < 1:10 or a minimum four-fold rise in HAI titer if the baseline titer is ≥ 1:10) for H1N1 and influenza B and H3N2 (by age group only) in the respective season's vaccine<br>b. Proportion of subjects with a seroprotective HAI titer (≥ 1:40) pre- and post-immunization at day 29 for each IIV antigen in the respective season's vaccine The geometric mean HAI titer (GMT) for each IIV antigen in the respective season's vaccine<br>c. The geometric mean HAI titer (GMT) for each IIV antigen in the respective season's vaccine

	Exploratory:<br>1. Associations between moderate/severe local and systemic reactogenicity events and quality of life outcomes in the full study population and by age group<br>2. Comparison between vaccination groups of proportion of participants with negative and positive perceptions of the vaccination experience based on responses to the Perceptions of Vaccination Experience questionnaire<br>3. Proportion of participants with difficulty performing adverse event monitoring based on responses to questions to the Perceptions of Vaccination Experience questionnaire<br>4. Mean injection site pain scores immediately after vaccination in both vaccination groups using the Faces Pain Scale<br>5. Compare the frequency and descriptions of immediate reactogenicity and immediate serious adverse events and adverse events of clinical interest between vaccination groups.<br>6. Associations between reactogenicity and demographic, co-morbidity and medication factors from subjects' medical histories, including statin use<br>7. Compare changes in local and systemic reactions in subjects who receive vaccine in both study years<br>8. Associations between HAI titers and moderate/severe local and systemic reactogenicity events.<br>9. Associations between HAI titers and demographic, co-morbidity and medication factors in subjects' medical histories including statin use<br>10. Comparison of seroprotection and seroconversion as defined by HAI titers and geometric mean HAI titers in subjects who receive vaccine in both study years<br>11. Comparison of seroprotection and seroconversion as defined by HAI titers and geometric mean HAI titers between 1 month and 6 months in the same subjects (Year 1 only)
**Estimated Time to Complete Enrollment:**	Approximately 6 months for enrollment during each of two consecutive flu seasons, for an overall duration of 18 months

1 BACKGROUND

1.1 Background

The Advisory Committee on Immunization Practices (ACIP) has long recommended influenza vaccination for older persons ≥65 years to prevent influenza and its complications[1]. In addition to standard inactivated influenza vaccine (IIV), several new licensed influenza vaccines are available for use in older adults, including FLUAD®' an adjuvanted trivalent inactivated influenza vaccine (aIIV3) and Fluzone® High-Dose IIV3 (IIV3-HD). The ACIP has not indicated a preferential recommendation for any of the influenza vaccine preparations approved for older adults[2].

Older adults are at high risk for influenza-related morbidity and mortality due to immunosenescence (decreased immune responses with age), multimorbidity, specific age-related diseases (e.g, congestive heart failure, chronic lung disease) and age-related changes in compensatory physiologic responses to inflammatory states[3-6]. Although the incidence of influenza is usually higher in younger adults and children than older adults, 90% of influenza-related deaths occur in older adults[7]. From 1976-2007, the estimated average number of annual influenza-associated deaths with underlying respiratory and circulatory causes in persons aged ≥65 years was 21,098 with a maximum number of 43,727[8]. The risk of hospitalization associated with influenza is highest in persons aged ≥65 years[9]. Influenza is also associated with functional decline, reduced quality of life, and disability in older adults[10].

Influenza vaccination is an important intervention to prevent influenza and its complications in older adults, but the immunogenicity and effectiveness of influenza vaccines are suboptimal in older adults due to immunosenescence[11]. Vaccine scientists have pursued different strategies to improve the immunogenicity and clinical effectiveness of influenza vaccine in older adults. One strategy is to increase the dose of hemagglutinin antigen in the vaccine[12]. The IIV3-HD has four times the dose of hemagglutinin antigen compared to standard-dose IIV3 (IIV3-SD). Compared with IIV3-SD, IIV3-HD is significantly more immunogenic for the influenza A strains and non-inferior for the B strain[13]. Studies have also demonstrated that the IIV3-HD is more effective in preventing influenza than IIV3-SD in older adults[14,15]. A large randomized controlled trial showed relative vaccine effectiveness of 24% for IIV3-HD compared with IIV3-SD, which equated to 4 fewer cases of laboratory-confirmed influenza per 1000 persons vaccinated after IIV3-HD compared with IIV3-SD[14]. Another strategy is to add an adjuvant to the influenza vaccine. The aIIV3 contains the MF59 adjuvant which is a squalene-based, oil-in-water emulsion[16]. Non-inferiority of aIIV3 compared with IIV3-SD was demonstrated for all three vaccine strains based on pre-defined thresholds for seroconversion rate differences and geometric mean titer ratios[13]. Observational studies conducted in Europe have found that aIIV3 is more effective in preventing influenza than IIV3-SD[16]. A confirmatory, randomized controlled clinical trial in the United States to verify and describe the clinical benefit of aIIV3 is required by the FDA under the accelerated approval regulations[17].

Clinicians and older adult patients now face an important choice between IIV3-SD, IIV-HD3 and aIIV3. The superior immunogenicity (for IIV-HD) and clinical effectiveness of IIV3-HD and aIIV3 may sway the choice towards these products compared with IIV3-SD, but the safety of these vaccines is a key factor in the decision. Although aIIV3, IIV-HD and IIV3-SD appear to have similar proportions of serious adverse events, aIIV3 and IIV3-HD have higher proportions of local and systemic reactions compared with IIV3-SD[15,18]. However, IIV3-HD and aIIV3 have not

been compared directly with regards to safety in the same trial. In separate clinical trials, the most common moderate/severe injection-site reaction was pain (4.2% aIIV3 and 4% IIV3-HD) and the most common moderate/severe systemic reaction was malaise/fatigue (3.5% aIIV3 and 6.3% IIV3-HD)[13,18]. These rates of moderate/severe reactions are higher than those reported for IIV3-SD. Moderate/severe reactions are generally those which limit or prevent usual daily activities. For older persons, the ability to independently perform basic activities of daily living (e.g., bathing, dressing, grooming, toileting, eating, mobility) and instrumental activities of daily living (e.g., cooking, housework, traveling, managing money, managing medications) are critical to quality of life. Any reaction that limits or prevent these activities will have a significant impact on health-related quality of life and is likely to be more clinically important than in younger, healthier populations[19-21].

1.2 Summary and Rationale

The safety profile or immunogenicity of aIIV3 and IIV3-HD have not been compared head-to-head. The decision of which vaccine to use in older adults will be enhanced by a better understanding of the safety and immunogenicity of these vaccines, as well as the relative impact on health-related quality of life, functioning and participant perspectives of the vaccination experience. Furthermore, the best method for collecting information on health-related quality of life, functional status and other non-medically attended adverse events after moderate to severe injection site or systemic reactions is not known. This study will address this gap in knowledge in older adults after vaccination. This study will also add value to pandemic influenza preparedness by providing data on risks and benefits of influenza vaccination in older adults that are not currently available.

2 STUDY OBJECTIVES AND OUTCOME MEASURES

2.1 Study Objectives

2.1.1 Primary Objectives:

1. To compare the proportions of moderate/severe injection-site pain after aIIV3 and IIV3-HD in the full study population
    a. Hypothesis: the proportion of subjects who have moderate/severe injection site pain within the first week post-vaccination will be non-inferior for aIlV3 compared to IIV3-HD in the full study population
2. To compare serious adverse events and events of clinical interest after aIIV3 and IIV3-HD in the full study population and by age-group
3. To compare the seroconversion rate for the H3N2 influenza A strain after aIIV3 and IIV3-HD in the full study population
    a. Hypothesis: the seroconversion rate for the H3N2 influenza A strain in the full study population after aIIV3 will be non-inferior to IIV-

2.1.2 Secondary Objectives

1. To compare the proportions of local and systemic reactions (other than moderate/severe injection site pain) after aIIV3 and IIV3-HD in the full study population and by age-group (65-79 years and ≥80 years)
2. To describe and compare changes in health-related quality of life after aIIV3 and IIV3-HD in the full study population and by age-group
3. To compare serum hemagglutination inhibition (HAI) antibody titers after aIIV3 and IIV3-HD for each of the three influenza vaccine strains contained in the respective vaccine for that season in the full study population and by age (except for seroconversion for the H3N2 strain in the full study population)

2.1.3 Exploratory Objectives:

1. To describe how the reactogenicity events affect health-related quality of life after IIV3 and IIV3-HD in the full study population and by age-group
2. To describe and compare participant perceptions about the vaccination experience
3. To describe participant perceptions of methods of adverse event monitoring in older adults receiving influenza vaccines.
4. To describe injection site pain immediately after vaccination after aIIV3 and IIV3-HD
5. To describe and compare the proportions of immediate adverse events and unsolicited adverse events after aIIV3 and IIV3-HD in the full study population and by age-group (65-79 years and ⩾80 years)
6. To describe factors (e.g., statin use) associated with reactogenicity to aIIV3 and IIV3-HD
7. To describe the safety of aIIV3 and IIV3-HD after repeat administration of the same product over 2 consecutive influenza seasons in a subset of subjects
8. To describe the relationship between reactogenicity and immunogenicity for aIIV3 and IIV3-HD
9. To explore factors (e.g., statin use) associated with immunogenicity for aIIV3 and IIV3-HD
10. To describe the immunogenicity of aIIV3 and IIV3-HD after repeat administration of the same product over 2 consecutive influenza seasons in a subset of subjects
11. To assess changes in serum hemagglutination inhibition at 1 month after aIIV3 and IIV3-HD vaccination, and at 6 months after vaccination in a subset of subjects

2.2 Study Outcome Measures

Where possible, all outcome measures will be evaluated and compared between the two treatment groups for the full population and in both age subsets (65-79 years and ≥80 years)

2.2.1 Primary Outcome Measures:

1. Comparison of the proportion of subjects reporting moderate/severe injection site pain within the first week post-vaccination in both treatment groups.

2. The frequency and descriptions of serious adverse events and adverse events of clinical interest observed in the two treatment groups.

3. H3N2 HAI seroconversion: The proportion of subjects achieving H3N2 seroconversion at day 29 (an HAI titer ≥ 1:40 at day 29 if the baseline titer is < 1:10 or a minimum four-fold rise in HAI titer if the baseline titer is ≥ 1:10) in the respective season's vaccine.

2.2.2 Secondary Outcome Measures

1. Comparison of local and systemic reactions within the first week post-vaccination in both treatment groups.
2. Change in scores on the Late Life Function & Disability Instrument (Year 1 only), EuroQOL 5 dimensions-5 level (EQ-5D-5L) and EuroQOL visual analogue scale (EQ VAS) pre-vaccination and post-vaccination will be compared between the vaccination groups and age groups.
3. HAI titers by vaccination group and age group (except for seroconversion to H3N2 strain for the full study population):
   a. The proportion of subjects achieving seroconversion at day 29 (an HAI titer ≥ 1:40 at day 29 if the baseline titer is < 1:10 or a minimum four-fold rise in HAI titer if the baseline titer is ≥ 1:10) for H1N1 and influenza B and H3N2 (by age group only) in the respective season's vaccine
   b. Proportion of subjects with a seroprotective HAI titer (≥ 1:40) pre- and post-immunization at day 29 for each IIV antigen in the respective season's vaccine
   c. The geometric mean HAI titer (GMT) for each IIV antigen in the respective season's vaccine

2.2.3 Exploratory Outcome Measures

1. Associations between moderate/severe local and systemic reactogenicity events and quality of life outcomes in the full study population and by age group
2. Comparison between vaccination groups of proportion of participants with negative and positive perceptions of the vaccination experience based on responses to the Perceptions of Vaccination Experience questionnaire.
3. Proportion of participants with difficulty performing adverse event monitoring based on responses to a questions to the Perceptions of Vaccination Experience questionnaire
4. Mean injection site pain scores immediately after vaccination in both vaccination groups using the Faces Pain Scale
5. Compare the frequency and descriptions of immediate reactogenicity and immediate serious adverse events and adverse events of clinical interest between vaccination groups.
6. Associations between reactogenicity and demographic, co-morbidity and medication factors from subjects' medical histories, including statin use
7. Compare changes in local and systemic reactions in subjects who receive vaccine in both study years
8. Associations between HAI titers and moderate/severe local and systemic reactogenicity events.
9. Associations between HAI titers and demographic, co-morbidity and medication factors in subjects' medical histories including statin use
10. Comparison of seroprotection and seroconversion as defined by HAI titers and geometric mean HAI titers in subjects who receive vaccine in both study years

11. Comparison of seroprotection and seroconversion as defined by HAI titers and geometric mean HAI titers between 1 month and 6 months in the same subjects (Year 1 only)

3 STUDY DESIGN

3.1 Main study design

This study is a prospective, randomized, blinded clinical trial to assess the safety of aIIV3 versus IIV3-HD in ≥440 adults age ≥65 years enrolled at Duke University Medical Center (Lead Contractor), ≥340 adults age ≥65 years enrolled at Boston Medical Center (BMC) (Contributing Contractor), and 100 adults age ≥65 years enrolled at Cincinnati (Contributing Subcontractor). Participants will be enrolled in 2017-18 (Duke and Boston) and 2018-19 (Duke, Boston, and Cincinnati) influenza seasons. Unblinded, licensed staff will perform vaccinations, and all other study personnel and subjects will be blinded throughout the study, with the exception of the Duke Project Manager, Boston Pharmacy, and Cincinnati Pharmacy staff (who have no involvement with study subjects). In addition, the study statistician will be blinded during data analysis. Older adults who have not received IIV during the respective influenza seasons during which they are recruited will be enrolled. Detailed health, demographic and health-related quality of life data will be collected from study participants at baseline prior to influenza vaccine receipt. With Day 1 serving as the day of vaccination, participants will be followed through Day 8 (total 8 days) for symptoms of reactogenicity as described in Section 5.4. Health-related quality of life and vaccination experience data will be collected during this time period. Participants will be followed through Day 43 for serious adverse events and adverse events of clinical interest, including health care utilization, as described in Sections 5.4 and 5.6.

3.2 Laboratory Studies

3.2.1 Influenza Hemagglutination Inhibition Assay

Participants will have blood draws on Day 1 (before vaccination) and Day 29 to be stored for serum hemagglutination inhibition (HAI) antibody titers. During 2017-18 influenza season (Year 1), a subset of approximately 100 patients at Duke will have a blood draw at Day 181 for an additional HAI titer. If funding is available, HAI antibody titers will be compared between groups receiving aIIV3 or IIV3-HD for each of the three influenza vaccine strains contained in the respective vaccines for that season. Additionally, if funding available, 60 year 1 participants will receive repeat immunization with the same vaccine in year 2 at Duke, and HAI titers will be performed on serum from Day 1 and Day 29. Participants will not receive individual HAI antibody titer results; these are not routinely used in clinical practice.

3.2.2 Future studies

Additional blood will be stored for further immune analyses depending on funding availability. Biologic specimens collected as part of this study and used in other studies examining the immune response to influenza vaccine will be linked to information (including identifying information) that participants provided in the current study. Participants are not expected to receive results of any future testing of their specimens.

4 STUDY ENROLLMENT AND WITHDRAWAL

4.1 Subject Inclusion Criteria

Subjects who meet all of the following criteria will be eligible to participate in this interventional study.

1. Persons aged ≥65 years, living in the community
2. Intention of receiving IIV vaccine based on ACIP-CDC guidelines
3. Willing to provide written informed consent prior to initiation of any study procedures
4. Able to speak English
5. Able and willing to complete baseline assessments and questionnaires, and to allow information to be collected from their electronic medical record
6. Able and willing to complete post-vaccine assessments and questionnaires independently or with assistance
7. Able and willing to have blood drawn for the study
8. Able and willing to return in about one month for a follow-up visit including completing questionnaires and having another blood test
9. Access to and ability to use a phone, independently or with assistance
10. Adequate vision and motor skills to complete the symptom diary form independently or with assistance.
11. Not living in a skilled nursing facility/nursing home/long term acute care facility

4.2 Subject Exclusion Criteria

Subjects who meet any of the following criteria will not be eligible to participate in this study:

1. Influenza vaccine receipt during the current influenza season prior to study enrollment
2. Enrolled in this study during the 2017-18 (Year 1) influenza season

   Note: Year 1 study participant will only be enrolled in Year 2 if they are participating in the sub-study on repeat vaccination
3. Has immunosuppression as a result of an underlying illness or treatment, or use of anti-cancer chemotherapy or radiation therapy within the preceding 12 months.
4. Has an active neoplastic disease (excluding non-melanoma skin cancer or prostate cancer that is stable in the absence of therapy) or a history of any hematologic malignancy*

   **Participants with a history of malignancy may be included if, after previous treatment by surgical excision, chemotherapy or radiation therapy, the participant has been observed for a period that in the investigator's estimation provides a reasonable assurance of sustained cure*
5. Thrombocytopenia, bleeding disorder, or anticoagulant use contraindicating intramuscular injection
6. Receipt of blood or blood-derived products in the past three months
7. History of febrile illness (≥ 100.0°F or 37.8°C) within the past 24 hours prior to IIV administration (temporary deferral)
8. Contraindication to IIV receipt including history of severe allergic reaction after a previous dose of any influenza vaccine; or to a vaccine component*, including egg protein; or a latex allergy

   **Formaldehyde, Octylphenol ethoxylate, neomycin, kanamycin, barium, cetyltrimethlyammonium bromide (CTAB)*
9. Any history of Guillain-Barré syndrome

10. Mild to severe dementia as determined by the Mini-Cog tool and the Rowland Universal Dementia Assessment Scale (RUDAS)
11. Substance use that could interfere with study compliance
12. Receipt of any inactivated licensed vaccine within 2 weeks, or live attenuated licensed vaccine within 4 weeks prior to enrollment in this study, or planning receipt of any vaccines during the 42 days post-vaccination period (including pneumococcal vaccines)
13. Receipt of Shingrix (Zoster Vaccine Recombinant, Adjuvanted) or HEPLISAV-B (Hepatitis B Vaccine (Recombinant), Adjuvanted) vaccine within 6 weeks prior to enrollment in this study, or planning receipt of Shingrix or HEPLISAV-B during the 42 days post-vaccination period.
14. Anyone who is already enrolled or plans to enroll in another clinical trial with an investigational product within 28 days of vaccine receipt. Co-enrollment in observational or behavioral intervention studies are allowed at any time while enrollment in a clinical trial involving an investigational product (other than vaccine) may occur after 30 days following vaccine receipt.
15. Hearing loss determined by the investigators to prevent successful communication over the phone
16. Any condition which, in the opinion of the investigators, may pose a health risk to the subject or interfere with the evaluation of the study objectives.
17. Anyone who is a relative or subordinate of any research study personnel.

4.3 Recruitment

Participants ≥65 years of age will be recruited from several sources at Duke University Medical Center (DUMC), Boston Medical Center (BMC), and Cincinnati using varying techniques. Study investigators will enroll at least 440 persons including at least 20% adults ≥ 80 years of age at Duke over two seasons (~220 participants per season), ~340 persons including at least 20% adults ≥ 80 years of age at Boston over two seasons (~170 per season), and ~ 100 persons including at least 20% adults ≥ 80 years of age at Cincinnati in 2018-2019 only. During 2017-2018 at Duke only, a subset of approximately 100 subjects will be assigned to receive an additional blood draw at day 181 (Section 5.2.1).

The general techniques for how subjects will be recruited include the following: Study staff, including PIs and study nurses, will approach their patients in clinic directly about the study during clinic visits; notify other health care professionals in their health system about the study via letters and flyers for potential referrals; notify potential subjects about the study via study registries and recruitment service programs; letters and talks to senior groups in various venues including Senior Centers and Continuing Care Retirement Communities; advertising in newspapers; flyers posted at senior locations; letters and talks to potential referral sources; and letter campaigns to older adults in the surrounding catchment area. More specific mechanisms to DUMC, BMC, and Cincinnati are described below.

At DUMC potential subjects will be approached via the following mechanisms: 1) Older participants of previous vaccine studies who have agreed to enroll in future studies; 2) Duke Center for Aging Human Subjects Registry, a unique long-standing registry of over 3000 individuals who volunteer for human studies; 3) Croasdaile and The Forest at Duke continuing care retirement communities. Medical care for these communities is provided by the Duke Division of Geriatrics, overseen by Dr. Schmader; 4) Duke's "Aging" network of senior centers; senior activities; and referrals from professionals who work with seniors cultivated via the work

of the Center for Aging; 5) Duke Geriatric Evaluation and Treatment Clinic (GET Clinic) and Duke University Health System primary care clinics. These sources are likely to be sufficient, but if not, we will utilize other past successful strategies such as a letter campaign to households of older adults and advertising in senior citizen newspapers and general local newspapers.

At BMC, recruitment will take place in the Boston Medical Center Geriatric Ambulatory Practice, during routine primary care visits and during dedicated influenza vaccine clinics. Information about the study will also be posted on the BMC Recruitment Services Program of the Clinical Translational Science Institute (ReSPECT) Registry, a source of research study information available to the BMC community. The BMC Clinical Research Resources Office may also help identify approaches to recruit subjects. We may also recruit older adults from outside the BMC system using databases available to the BMC investigators, or advertisements in local newspapers known to carry information about research studies recruiting participants.

At Cincinnati (CCHMC), recruitment will take place at Maple Knoll Village, a retirement community with over 800 older adults living independently and the greater Cincinnati community through the CCHMC Office of Clinical Trials (OCTR). Participants will be recruited via Lunch and Learn Seminars, the resident newsletter, the community e-newsletter, information sessions at their happy hours, direct approach pieces in their mailboxes, employee email, CCHMC flyer boards and OCTR approved social media postings. Subjects will conduct study visits either at the clinic within Maple Knoll Village or at the Schubert Research Clinic at CCHMC.

At all sites, existing patients may be approached by phone call. The study will be reviewed with the patient, and if the patient is interested, then initial eligibility screening will take place on the phone following an IRB-approved script. A waiver of documentation of consent will be approved from the reviewing IRB in order to carry out these screening activities.

A subset of approximately 60 subjects enrolled and vaccinated in the first year of the study at Duke will be included in a repeat vaccination sub-study in the second year of the study. The sub-study will explore the safety and immunogenicity of repeat vaccination with the same vaccine product in the subsequent influenza season. The study enrollment procedures will be the same, with the following modifications: At the time of enrollment in the first year, the subjects will be asked in sequential order if they would like to participate in a repeat vaccination sub-study during the next season. The first subjects enrolled at Duke (up to n=60), who express interest in this repeat vaccination sub-study will be included. Subjects need not consent to this part of the study to participate in the main study. In the first year, this convenience sample of sub-study subjects will undergo the same randomization procedures as other subjects in section 5.2.1. The subjects will complete procedures for the first study influenza season as in section 5.1. Contact information will be maintained, and subjects will be contacted and reminded to return to the study center in September/October the next influenza season. Upon return for the second year of the study, subjects will be consented for the sub-study and screened to see if they continue to meet the study eligibility criteria. In year 2, these subjects will receive the same vaccine product as in year 1, using the formulation licensed by FDA for the year 2 season; influenza strains included in the vaccines may be different in the consecutive years. These subjects will remain blinded to the vaccine product in Year 2. Year 1 study participants will only be enrolled in Year 2 of the study if they consent to and are still eligible for participating in the sub-study on repeat vaccination.

4.4 Reasons for and Handling of Withdrawals

The following may be reason for study withdrawal:

- As deemed necessary by the principal investigator (PI).
- Subject withdrawal of consent.
- Loss to follow-up.
- Subject unable to return for study appointments
- Termination of the study by the sponsor.
- Subjects not meeting eligibility criteria in the repeat vaccination study will be withdrawn at the time this is determined

Subjects may withdraw their consent for study participation at any time and for any reason, without penalty. Subjects who withdraw from the study prior to randomization will be replaced. Subjects who withdraw from the study after randomization will not be replaced. Data collected before withdrawal will still be used for analysis.

4.5 Termination of Study

This study may be terminated for safety concerns of the principal investigators from the Lead or Contributing sites, CDC, or participating Institutional Review Boards (IRBs).

5 STUDY SCHEDULE, PROCEDURES, & EVLAUTIONS

5.1 Schedule of events and data collection

Persons meeting the proposed eligibility criteria (Section 4) will be recruited. Written informed consent (Appendix A) will be obtained from study participants prior to conducting any study procedures. Prescreening may take place over the phone prior to Visit 1. Table 1 describes the schedule of study visits with further details below.

Table 1. Visit Schedule

**Procedure**	**Visit 1 Day 1**	**Visit 2 Day 3 + 2**	**Unscheduled Visit**	**Visit 3 Day 9 + 3**	**Visit 4 Day 29 ± 7**	**Visit 5 Day 43 + 7**	**Visit 6 Day 181 + 14[a]**
Type of contact	Clinic	Phone or Clinic	Clinic	Phone or Clinic	Clinic	Phone or Clinic	Clinic
Informed consent & Medical Release of Information	X						
Review Eligibility Criteria	X						
Cognitive Assessment	X						
Sociodemographics	X						
Medical history	X	X	X	X	X	X	X
Medications, including statins	X	X	X	X	X	X	X
Influenza Vaccination History	X						
Vital signs/temperature	X		X		X		X
Body mass index	X						X
Obtain health-related quality of life assessment	X			X[e]			

Procedure	Visit 1 Day 1	Visit 2 Day 3 + 2	Unscheduled Visit	Visit 3 Day 9 + 3	Visit 4 Day 29 ± 7	Visit 5 Day 43 + 7	Visit 6 Day 181 + 14[a]
Participant perception of vaccination	X[d]			X[d]	X[d]		
Baseline reactogenicity	X[c]						
Venipuncture	X				X		X
Randomization	X[d]						
Vaccination	X						
Diary & supplies	X						
Assess for injection site pain at least 15 min. after immunization	X						
At ≥ 15 min. assess for any immediate reactogenicity symptoms	X						
Obtain solicited adverse events		X	X[b]	X			
Obtain unsolicited adverse events		X	X	X	X	X	
Obtain SAE information, AEs of clinical interest, and new onset medical conditions		X	X	X	X	X	
Obtain health care utilization data		X	X	X	X	X	

[a]For subset of approximately 100 subjects receiving third blood draw 6 months post-vaccination in 2017-18
[b]For unscheduled visits, solicited AEs will be collected only for days ≤9
[c]Baseline reactogenicity will be performed before venipuncture and vaccination
[d]Does not apply to subjects participating in the Year 2 repeat vaccination during study Year 2
[e]Will collect Day 3 and Day 9 health related quality of life instruments (EQ-5D-5L and Vaccine Reaction Questionnaire) on V3 phone call

Visit 1, Study Day 1 - Screening, Enrollment, and Vaccination (Clinic Visit)

- Obtain written informed consent and release of medical record information
    - Note: In Year 2, subjects participating in the sub-study on repeat vaccination will sign a new consent form
- Determine if subject will participate in long term immunogenicity sub-study or repeat vaccination sub-study (Year 1 only)
- Review and confirm study eligibility
- Perform cognitive assessment with the Mini-Cog tool[22] (and RUDAS if needed)(Appendix B,C)
    - Scores 3-5 on MiniCog will be eligible
    - Scores 0-1 on MiniCog will be ineligible
    - Scores of 2 on MiniCog: person will undergo further screening with RUDAS. If person scores 23 or higher (range = 0-30), they will be eligible.
    - Potential participants may have low scores on the cognitive screening tests and be ineligible for the study. In this case, the study doctor or designee will review the results with the individual and recommend follow-up with the individual's health care provider.

- Obtain information on preferred method of contact for follow-up (telephone or email reminder), and obtain contact info for caregiver/significant other
- Obtain demographic data
    - Age, gender, race/ethnicity, language spoken, contact information, education, insurance payer, employment status, living alone or not
- Obtain medical history including chronic conditions, hearing and sensory impairment, chronic pain
- Obtain concomitant medications, including use of statin medications.
- Obtain influenza immunization history for the previous two seasons (identify whether vaccine used was FLUAD, Fluzone-High Dose, some other inactivated influenza vaccine, or unknown, and indicate if this information came from the patient or their chart)
- Obtain vital signs including oral temperature, blood pressure, and pulse; and height and weight in order to calculate body mass index (BMI)
- Obtain baseline health-related quality of life assessments prior to vaccination (Section 5.5)
- Obtain answers to first two questions of perceptions of the vaccination experience questionnaire
- Perform pre-vaccination baseline immediate reactogenicity assessment (Appendix D)
- Obtain one tube of blood (~10 mL) prior to vaccination for serologic analysis (Section 5.7.1)
    - If unable to draw blood, subject will remain in study
    - If less than 1 mL of processed serum is collected, it is a protocol deviation
- Randomize study participant to aIIV3 or IIV3-HD administration (Section 5.2.1)
    - Subjects in the second year of the repeat vaccination sub-study will be assigned to receive the same vaccination product that they received the first year (using the FDA-formulation for the year 2 season)
- Administer assigned study products – Unblinded trained, licensed staff will administer either aIIV3 or IIV3-HD as described in Section 5.3.1. Ensure participants receive inactivated influenza Vaccine Information Sheets (VIS) during visit. (https://www.cdc.gov/vaccines/hcp/vis/vis-statements/flu-largetype.pdf) Participants and study staff doing follow-up evaluations are to remain blinded.
- Dispense symptom diary (Appendix E), paper version of EQ-5D-5L quality of life tool, oral digital thermometer with large display, and injection site measurement tool with predetermined local reaction measurement scales. **Review instructions for use of thermometer, injection site reaction measurement tool, diary completion, and EQ-5D-5L. Encourage participants to complete diary at the same time every evening. Should a subject misplace the study-provided thermometer, any oral thermometer can be used for symptom diary reporting.**
- Assess for immediate injection site pain using the Faces Pain Scale (Appendix F), at ≥ 15 minutes after vaccination
- At ≥ 15 minutes after vaccination assess for any immediate reactogenicity symptoms and other adverse events using the assessment form used for baseline reactogenicity (Appendix D)
- Confirm preferred method of contact for follow-up (telephone or email reminder)
- Confirm date of next appointment

Study Days 1 – 8

Participants complete symptom diary form and FACES pain scale (injection site pain) on days 1-8 and complete the paper version of EQ-5D-5L on Day 3.

Visit 2, Study Day 3 (window Days 3 – 5) Phone Call or Clinic Follow-Up

Study staff will contact study participants to review and record the following:

- Diary data and any solicited and unsolicited AEs, SAEs, adverse events of clinical interest, any new medical conditions, and any change in medications as described in Section 5.4.
- Health care utilization data (Sections 5.6).
- Participants will be reminded to complete diary card, and the health related quality of life questionnaires, EQ-5D-5L and Vaccine Reaction Questionnaire at home and that they will be contacted again after Day 8. Study team members have the option to complete the EQ-5D-DL and/or Vaccine Reaction Questionnaire over the phone or in person with the subject
- Should this visit not occur or occur out of window, it will not be a protocol deviation

Visit 3, Study Day 9 (window Days 9 – 12) Phone Call or Clinic Follow-Up

Study staff will contact study participants to review and record the following:

- Diary data and any solicited and unsolicited AEs, SAEs, adverse events of clinical interest, any new medical conditions, and any change in medications as described in Section 5.4.
- Health-related quality of life assessment administered by study personnel (Section 5.5)
- Participant perception of vaccination experience (Section 5.5.3)
  - Participant experience will not be recorded for subjects in the second year of the repeat vaccination sub-study.
- Collect responses from the Day 3 EQ-5D-5L and Vaccine Reaction Questionnaire
- Health care utilization data (Sections 5.6).
- Participants who are lost to follow-up by phone contact will be called weekly until contact made or until approximately Day 47, whichever comes first, following vaccination for safety assessment.

Visit 4, Study Day 29 (window Days 22 – 36) Clinic Visit

- Obtain vital signs including oral temperature, blood pressure, and pulse
- Record any unsolicited AEs, SAEs, adverse events of clinical interest, any new medical conditions, and any change in medications (Section 5.4)
- Participant perception of vaccination experience (Section 5.5.3)
  - Participant experience will not be recorded for subjects in the second year of the repeat vaccination sub-study.
- Record health care utilization data (Section 5.6.)
- Obtain one tube of blood (~10 ml) for serologic analysis (Section 5.7.1)
  - If less than 1 mL of processed serum is collected, it is a protocol deviation
- Confirm preferred method of contact for follow-up (telephone or email reminder)

Visit 5, Study Day 43 (window Days 43 – 50) Phone Call or Clinic Follow-up

- Record any unsolicited AEs, SAEs, adverse events of clinical interest, any new medical conditions, and any change in medications (Section 5.4)
- The study team will follow-up on any SAEs to the extent possible that occurred during the 43-day study period, even if follow-up occurs after the Day 43-50 window.

- Record health care utilization data (Section 5.6.)

Visit 6, Day 181 (window Days 181-195) Clinic Visit for immunogenicity subset at Duke during 2017-2018 season only, (n ≥ 100)

- Review changes in medical history and medications and AEs/SAEs
- Obtain vital signs and BMI
- Obtain one tube of blood (~10 ml) from subset of approximately 100 subjects for serologic analysis (Section 5.7.1)
    - If less than 1 mL of processed serum is collected, it is a protocol deviation

Unscheduled Visits associated with study/vaccine (≤43 days)

- Obtain vital signs including oral temperature, blood pressure, and pulse
- Record any solicited (≤ day 9) AEs, unsolicited (≤ day 43) AEs, SAEs, adverse events of clinical interest, new onset chronic medical conditions, and concomitant medications (≤ day 43) as described in Section 5.4
- Obtain health care utilization data (Section 5.6) (≤ day 43)
- Confirm preferred method of contact for follow-up (telephone or email reminder)

End of Study

After unblinding, study staff will contact subjects to inform them which vaccine they received. Study staff will also inform primary care providers which vaccine their patient received. Subjects will be provided with a letter thanking them for their participation and a plain language summary of the main results of the study when available. The main results will not be provided to participants earlier than public release of the findings.

5.2 Treatment Assignment Procedures

This study is a prospective, randomized, blinded clinical trial involving subjects aged ≥65 years of age who are to receive IIV vaccines.

5.2.1 Randomization

Participants aged 65 to 79 will be randomized (1:1) to receive either aIIV3 or IIV3-HD using a permuted block randomization scheme stratified by Lead and Contributing Site(s) for a total of ≥704 subjects. In Year 1 there will be 2 sites (Duke and Boston) and in Year 2 there will be 3 sites (Duke, Boston, and Cincinnati). A separate permuted block will be allocated for ≥176 subjects who are age 80 or older, thus getting an overall N≥880. Additionally, the first ≈100 patients at Duke who opt-in will be assigned to return on day 181 for an additional blood draw. The repeat vaccination sub-study in year 2 will use a convenience sample and will not require a 1:1 distribution of subjects into the two study arms, and may include Duke subjects in the waning immunity sub-study (i.e., those that consented to blood draw at Study Day ≥181). The project statistician will generate permuted block randomization schemes that will be uploaded to the Research Electronic Data Capture (REDCap) study database (Section 7.2). The randomization schedule will not be available to the study staff, so the next randomization allocation will not be known before randomization occurs. Following confirmation of study eligibility criteria during Visit 1, participant randomization will be through REDCap with treatment allocation recorded on the case report form (CRF) by unblinded research staff who administer the vaccine. Participants and study staff doing follow-up evaluations will not have access to the treatment allocation CRF to maintain blinding. In the event that REDCap is unavailable, manual

randomization will occur through the use of envelopes. The project statistician will prepare 20 envelopes per age group per site (total of 40 per site) that will use the same randomization strategy as the primary scheme embedded in REDCap. When an unblinded team member is informed of the age group, he/she will pull the next envelope in order. In order to capture the allocation per subject, a separate form in REDCap will be used by the unblinded personnel to add the assignment. A log will need to be kept at the site capturing these instances.

5.3 Data Collection

5.3.1 Vaccine Supply, Storage, Administration, and Blinding

In order to ensure adherence to study randomization assignment, licensed aIIV3 FLUAD® (Seqirus) and IIV3-HD Fluzone® High-Dose (Sanofi) vaccines will be administered as study procedures. FLUAD® and Fluzone® High-Dose vaccines (prefilled syringes) will be purchased for study administration and maintained at the respective study locations and stored at 2° to 8°C in a research-specific medication refrigerator according to package insert specifications and site-specific standard operating procedures (SOPs). While research staff maintain daily temperature logs for the medication refrigeration, it is also monitored 24/7 with alarm activation if out of range. Research staff are notified of any alarm activations and have an on-call system in place to report to the research center for further investigation. Any potentially compromised vaccine will be quarantined for further disposition based on site-specific standard operating procedures (SOPs) and investigator assessment.

A single administration of both aIIV3 and IIV3-HD comprises intramuscular delivery of 0.5mL total volume of each vaccine for adults. Vaccine type, lot number, dosage, and site of vaccine administration will be recorded by research staff. There is a visual difference between aIIV3 and IIV3-HD, therefore the study will involve personnel who are blinded to the treatment allocation and data analysis and different personnel who are unblinded to treatment allocation.

- Emergency management supplies will be available for initial treatment of an allergic reaction, if needed.

<u>The following groups will be blinded to treatment allocation:</u>
• Investigational site staff performing data collection and analysis including the Investigator
• Participants

<u>The following study personnel will be unblinded to treatment allocation:</u>
• Investigational site staff involved in preparation and administration of the study vaccines. Unblinded personnel may also assist in pre-vaccination assessments of study subjects.

aIIV3 and IIV3-HD will be administered in blinded participants in the deltoid, preferably in the non-dominant arm by unblinded licensed staff. In order to keep the participant blinded, the vaccine administrator will keep the prefilled syringes out of view of the participant at all times and will instruct the participant to turn their head in the opposite direction of the arm in which the vaccine is being administered. Similar instructions will be given to any persons accompanying the patient in the room. After administration, used study syringes will be disposed of according to site-specific SOPs. A licensed provider (MD, NP, PA, DO, RN), who will be trained on treating adverse reactions, will be present and immediately available at the time of vaccine administration along with emergency management supplies available for initial treatment of an

allergic reaction if needed. Additionally, clinical members of the blinded data collection team will be present to assist study subjects.

The participant and primary care provider will receive documentation of receipt of influenza vaccine without specification of whether it was high dose or adjuvanted vaccine to preserve blinding. In the event of individual participant clinical safety issues or overall study safety concerns, then blinding may be broken

5.4 Reactogenicity and Safety Assessment

Participants will have an assessment of well-being and reactogenicity symptoms at baseline (pre-vaccination) and will be assessed for any immediate reactogenicity or other unsolicited adverse events at least 15 minutes after vaccine administration while at the study site. They will be assessed for medical symptoms (e.g., fatigue, malaise, myalgia, arthralgia, headache, nausea) at baseline and at ≥15 minutes after vaccination using the assessment form in Appendix D. They will be assessed for any potential injection site reactions including tenderness, pruritus, ecchymosis, erythema or induration at baseline and at ≥15 minutes following vaccination. They will also be assessed for potential systemic reactions such as anaphylaxis related symptoms (e.g., dyspnea, chest tightness, wheezing, cough, stridor, urticaria, flushing, nasal congestion, dizziness, syncope, diaphoresis, emesis) immediately following vaccination until discharge. The severity of any reaction will be graded according to the severity ratings in Tables 3 and 4. Injection site pain severity will be assessed at ≥15 minutes after vaccination using the FACES pain scale[23] with 0-10 numeric rating, with 4-6 being moderate pain and 7-10 being severe pain (Appendix F).

In addition to immediate post-vaccination assessments, the occurrence of adverse events [including SAEs (Section 5.4.2), solicited reactogenicity events, and unsolicited adverse events (including events of clinical interest (Section 5.4.1.1)], new onset of medical conditions, concomitant medication use, and unscheduled medical care will be assessed daily through post-vaccination Day 8 using a standard symptom diary. Participants will also be instructed to self-report worst pain at the injection site in the past 24 hours using the Faces Pain Scale through post-vaccination Day 8. At the time of immunization, participants will be given a thermometer and injection site reaction measurement aid and instructed on using the symptom diary to document oral temperatures and post-injection symptoms. Beginning on the evening of Study Visit 1 (Day 1) following vaccination, participants will record their oral temperature using the study-supplied thermometer, the occurrence of AEs, and concomitant medication use for Days 1 – 8. Temperature will be recorded at roughly the same time each day. If a temperature ≥ 99.5°F (37.5°C) is recorded, a second measurement will be taken. If more than one temperature is taken on the same day, the highest temperature should be recorded. Participants will be queried during Visits 2 and 3 to review solicited injection site AEs that occurred each day, with the help of the information they have recorded in the symptom diary. These AEs will be classified as mild, moderate, or severe based on the criteria used for licensure of Fluzone® High-Dose and FLUAD™ and as described in Table 2**.**

Table 2. Solicited Injection-site Reactogenicity

Symptom	Mild (Grade 1)	Moderate (Grade 2)	Severe (Grade 3)
**Pain**	Noticeable with no limitation in normal daily activity	Some limitation in normal daily activity	Completely unable to perform normal daily activity
**Tenderness**	Noticeable with no limitation in normal daily activity	Some limitation in normal daily activity	Completely unable to perform normal daily activity
**Shoulder Pain on Side of Vaccination**	Noticeable with no limitation in normal daily activity	Some limitation in normal daily activity	Completely unable to perform normal daily activity
**Induration/ Swelling**	Any to <25 mm	≥25 to <50 mm	≥50 mm
**Erythema/ Redness**	Any to <25 mm	≥25 to <50 mm	≥50 mm

Participants will also be queried during Visit 3 on common post-injection systemic symptoms as described in Table 3. Participants will also be encouraged to report any significant unsolicited adverse events in an open-ended question format, e.g. "How are you doing? Are you having any new issues since we saw you last? If so, please tell me about them." Unsolicited adverse events will be graded as described in Table 4. Participants who report severe solicited adverse events or express any concern about symptoms/unsolicited events will be encouraged to follow up with their primary care provider. Study staff will assist with coordination of referral appointments as necessary. Medical records will be obtained and reviewed for any unscheduled medical appointment through post-vaccination Day 43.

Table 3. Solicited Systemic Reactogenicity

Systemic	Mild (Grade 1)	Moderate (Grade 2)	Severe (Grade 3)
**Fever** (° C)<br>(° F)	≥37.5 - ≤38.0<br>≥99.5 - ≤100.4	>38.0 - ≤39.0<br>>100.4 - ≤102.2	>39.0<br>>102.2
**Chills**	Noticeable with no limitation in normal daily activity	Some limitation in normal daily activity	Completely unable to perform normal daily activity
**Fatigue**	Noticeable with no limitation in normal daily activity	Some limitation in normal daily activity	Completely unable to perform normal daily activity
**Malaise**	Noticeable with no limitation in normal daily activity	Some limitation in normal daily activity	Completely unable to perform normal daily activity
**Myalgia**	Noticeable with no limitation in normal daily activity	Some limitation in normal daily activity	Completely unable to perform normal daily activity
**Arthralgia**	Noticeable with no limitation in normal daily activity	Some limitation in normal daily activity	Completely unable to perform normal daily activity
**Nausea**	Noticeable with no limitation in normal daily	Some limitation in	Completely unable to

Systemic	Mild (Grade 1)	Moderate (Grade 2)	Severe (Grade 3)
	activity	normal daily activity	perform normal daily activity
**Vomiting**	Noticeable with no limitation in normal daily activity	Some limitation in normal daily activity	Completely unable to perform normal daily activity
**Diarrhea**	Noticeable with no limitation in normal daily activity	Some limitation in normal daily activity	Completely unable to perform normal daily activity
**Headache**	Noticeable with no limitation in normal daily activity	Some limitation in normal daily activity	Completely unable to perform normal daily activity

Table 4 Unsolicited Adverse Events

Systemic Illness	Mild (Grade 1)	Moderate (Grade 2)	Severe (Grade 3)
**Illness or clinical adverse event**	Noticeable with no limitation in normal daily activity	Some limitation in normal daily activity	Completely unable to perform normal daily activity

We will monitor study participants for the development of new onset acute or chronic medical conditions during the protocol-defined surveillance period of 42 days post-vaccination. These AEs will be reviewed periodically by a safety monitoring panel (Section 5.4.3). We will monitor study participants for SAEs during the protocol-defined surveillance period of 42 days post-vaccination and up to 181 days for the longer-term immunogenicity subset (Section 5.4.2).

5.4.1.1 Adverse events of clinical interest

We will also collect information regarding the occurrence of any adverse events of clinical interest. Events of clinical interest include syncope during post-vaccination monitoring in clinic, anaphylaxis in the first 24 hours after immunization, and new onset immune-mediated disease as defined in Appendix G.

5.4.1.2 Causality (relatedness) Assessment

Study site investigators will assess relatedness to vaccine or study procedures (related, possibly related, unlikely related, or not related) for SAEs, AEs, and AEs of clinical interest. Relatedness determinations of these events will inform IRB reporting and safety monitoring (Section 5.4.3). Solicited symptoms in Table 3 will all be considered to be related to vaccine and causality assessment will not be done for these events. The study investigators will use their clinical judgement to make causality assessments and may consult the Expert Safety Panel or CISA Project for assistance with causality determinations. The final causality assessment decision is the responsibility of the site PI where the subject was enrolled.

5.4.2 Reporting of Adverse Events

An adverse event (AE) is any untoward medical occurrence in a patient or clinical investigation subject administered a pharmaceutical product and which does not necessarily have to have a causal relationship with this treatment.

If indicated, AEs occurring during the study will be reported to the Vaccine Adverse Event Reporting System (VAERS). The National Childhood Vaccine Injury Act requires healthcare providers to report the following AEs to VAERS:

- Any adverse event listed by the vaccine manufacturer as a contraindication to further doses of the vaccine; or
- Any adverse event listed in the VAERS Table of Reportable Events Following Vaccination [PDF - 75KB] that occurs within the specified time period after vaccination.

In addition, CDC encourages reporting of any clinically significant adverse event that occurs in a patient following a vaccination, even if there is uncertainty regarding if a vaccine caused the event.

A serious adverse event (SAE) is defined as an AE that meets one of the following conditions[24]:

- Results in death
- Is life-threatening (defined as immediate risk of death at the time of the event)
- Requires inpatient hospitalization (initial or prolonged)
- Results in a persistent or significant disability/incapacity
- Any other important medical event that may not result in death, be life threatening, or require hospitalization, may be considered an SAE when, based upon appropriate medical judgment, the event may jeopardize the subject and may require medical or surgical intervention to prevent one of the outcomes listed above.

SAE and AE reporting will occur consistent with institutional policy. The original verbatim terms used by investigators to identify SAEs and adverse events of clinical interest in the case report form will be mapped to preferred terms using the Medical Dictionary for Regulatory Activities (MedDRA) dictionary (http://www.meddra.org/).

Vaccine-related SAEs will be medically attended per routine care

SAEs will be reported promptly to the overseeing IRBs in accordance with institutional procedures. Any unanticipated problems resulting from study conduct related to participation will be reported promptly to the reviewing IRBs and CDC, in accordance with institutional procedures.

The duration of subject’s participation in the study varies based on their involvement in the immunogenicity sub-study. Therefore, the period for monitoring and reporting SAEs varies among subjects as shown below:

- Subjects NOT participating in long-term immunogenicity subset: duration of study participation is 42-days post vaccination
- Subjects participating in long-term immunogenicity subset: duration of study participation is 181 days post-vaccination

The study will report only SAEs occurring during each subject's participation in the study.

More information on potential risks and benefits is located under Human Subjects, section 8.3.

5.4.3 Safety Monitoring Plan

Although FLUAD® and Fluzone® High-Dose are licensed vaccines, there is less US safety experience with these vaccines than with standard influenza vaccines, particularly for FLUAD®, which was recently licensed in the United States (November 24, 2015). Fluzone® High Dose was licensed on December 23, 2009 in the United States. Also, less data are available in persons ≥80 years or for those with mild cognitive impairment than in younger, healthier adults. This is the first study conducted in the CISA Project where at least one death is somewhat likely to occur during the study period in a study participant (due to the older age-group of the participants). Therefore, the goal of the safety monitoring plan is to protect the health of the study population and ensure adequate communication of potential risks, and to provide situational awareness of potential safety signals from this study to CDC Immunization Safety Office (ISO) leadership. This plan is designed to monitor safety while minimizing introduction of bias into the study and minimizing burden to study investigators. The safety monitoring plan is described in Appendix H.

5.5 Health-Related Quality of Life (HRQOL)

5.5.1 Generic Measures of HRQOL

Late-Life Function & Disability Instrument (LLFDI) and Late-Life Function & Disability Instrument – Computer Adaptive Test (LLFDI-CAT) (Year 1 Only)

The Late-Life FDI is an HRQOL instrument for assessing function (ability to perform discrete actions or activities as part of daily routines) and disability (socially-defined tasks) in community-dwelling older adults[25-27]. The function component is a 32-item questionnaire that assesses how much difficulty a person has doing a range of upper extremity, basic lower extremity, and advanced lower extremity functions. The response categories for the function instrument are none, a little, some, quite a lot, cannot do in relation to the question, 'How much difficulty do you have?'. The disability component is a 16-item questionnaire that assesses how frequently a community dwelling older adult performs a particular task and the extent to which they feel limited in doing the task. The response categories for the disability instrument are very often, often, once in a while, almost never, never in response to 'how often do you . . ?' and not at all, a little, somewhat, a lot, completely in response to 'to what extent do you feel limited in?'

Each instrument generates a raw score, scaled score, and standard error for each dimension. The function component displays a total function score as well as separate scores for upper extremity, basic lower extremity, and advanced lower extremity subscales. The disability component displays a frequency total score, with social role and personal role subscale scores, and a limitation total score, with instrumental role and management role subscale scores. The scores range from 0-100 with high scores indicating higher levels of functioning and ability.

For the function section, the minimum clinically important difference ranges from 2.7 – 4.3. The disability section is split into two dimensions: limitations and frequency in doing a task. For limitations, the minimum clinically important difference is 16.7. For frequency, the minimum clinically important difference is 7.8.

The Late-Life Function & Disability Instrument – Computer Adaptive Test (LLFDI-CAT) is a modification of the LLFDI that expands the number of items and incorporates the World Health Organization's International Classification of Functioning, Disability Health domains of activity limitations (function) and participation restriction (disability)[28] (Appendix I). The Computer Adaptive Test method significantly reduces administration time and respondent burden. The LLFDI-CAT uses the same response categories as the LLFDI and generates similar summary and subscale scores

These instruments have several advantages for use in this study. They provide measures of extremity function and multiple activities for community dwelling older adults. They are validated, reliable, and responsive in older populations and various diseases. They are designed for self-completion. They employ a standardized scoring system, and are free of charge.

EQ-5D

The EQ-5D is a standardized, generic measure of health status that provides information on health-related quality of life and activities of daily living relevant to older adults: mobility, self-care, usual activities, pain/discomfort and anxiety/depression (http://www.euroqol.org/)[29]. In addition, the instrument contains the EQ Visual Analogue Scale (EQ-VAS) which measures the respondent's self-rated health.

The EQ-5D-5L is the new version of the EQ-5D that increases the levels of severity from three to five to significantly increase reliability and sensitivity while maintaining feasibility and reducing ceiling effects (Appendix J)[30,31]. The descriptive system comprises 5 dimensions of mobility, self-care, usual activities, pain/discomfort, anxiety/depression. For each of these dimensions, there are 5 response levels: no problems, slight problems, moderate problems, severe problems, and extreme problems. The respondent is asked to indicate his/her health state by ticking in the box against the most appropriate statement in each of the 5 dimensions. This decision results in a 1-digit number expressing the level selected for that dimension. The digits for 5 dimensions can be combined in a 5-digit number describing the respondent's health state from 11111 as best health and 55555 as worst health These numbers are converted to a Utility Index that ranges from -0.109 (worst health) to 1.000 (best health) for US specific values. The minimum clinically important difference ranges from 0.05 to 0.1 depending on health conditions being studied. The EQ VAS records the respondent's self-rated health on a 20 cm vertical, visual analogue scale with endpoints labelled 'the best health you can imagine' (100) and 'the worst health you can imagine' (0). The respondent marks an 'X' on the scale number to indicate how their health is 'today.' The minimum clinically important difference on the VAS is 8.

The EQ-5D-5L, EQ-VAS have several advantages for use in this study. The measure is applicable to a wide range of health conditions and treatments and provide a simple descriptive profile and a single index value for health status. It has been validated in US and international populations and in older adults[29,32,33]. The measure is useful for monitoring the health status of patient groups at different moments in time and assessing the seriousness of conditions at

different moments in time. The measure is designed for self-completion by respondents. It is simple, straightforward, take only a few minutes to complete and can easily be completed by older adults. The instrument was designed to reduce respondent burden while achieving standards of precision for purposes of group comparisons involving multiple health dimensions. It has been widely used throughout the world in many different studies, including randomized controlled clinical trials, vaccine studies, and health-related quality of life studies in older adults.

5.5.2 Vaccination Specific Measure of HRQOL

An important issue with the proposed health status instruments is that they are generic measures of HRQOL. Therefore, the instruments are sensitive to anything that occurs in a participant's life and are not specific to vaccine reactogenicity We will employ a measure that relies on the concept of vaccine reactogenicity specific interference with activities and impact on HRQOL [34,35].

The vaccination reaction specific measure will consist of specific instructions and interference with daily living items using a five-category word response as "not at all"; "a little"; "somewhat"; "a lot"; or "completely" (Appendix K). The participant would be asked to "circle the response that best describes how these problems from your flu shot have made things harder to" followed by key functional activities for older adults [34,36].

5.5.3 Perceptions of the Vaccination Experience and Methods of Adverse Event Monitoring (Not Applicable for Repeat Vaccination Sub-Study)

The participant's perception of the vaccination experience and methods of adverse event monitoring will be collected with a questionnaire that includes their preferences, values and knowledge about vaccination. The participant's perceptions about their participation in the vaccine clinical trial will be assessed with a questionnaire as well (Appendix L).

5.6 Health Care Utilization

Participants will be asked to report health care utilization including: telephone calls to the medical provider for medical advice, e-mail portal, electronic health record, clinic visits, urgent care visits, emergency department visits and hospital admissions occurring through day 43 according to the schedule in Table 1 above. The reason for health care use will also be obtained. Electronic or paper health records will be obtained and reviewed to confirm reports of clinic visits, urgent care visits, emergency department visits and hospital admissions. Health care utilization and the reason for health care utilization will be recorded on the symptom diary.

5.7 Biospecimens Collection & Handling

5.7.1 Serum

Blood specimens will be collected during study visits as described in Table 1.
All blood samples (≈10 mL) will be collected into serum separator tubes and processed as follows:

- Allow blood to clot at room temperature for at least 30 minutes while standing upright in a rack.
- Centrifuge tube within 8 hours of collection at 1100 to 1300 RCF(g) for 10 minutes.
    - Gently remove the vacutainer stopper avoiding serum contamination with red blood cells. Using a single-use pipette, transfer 1.0 mL aliquots of serum (top layer) into 1.0mL or 1.8 mL cryovials, up to 5 cryovials are expected. If less than 1 mL of processed serum is collected, it is a protocol deviation

- All cryovial aliquots will be barcode labelled and contain a unique identifier via REDCap. Numbers should be placed lengthwise on the tube.
- Freeze the cryovials at -80°C in the temperature-monitored research center freezer for future shipment.
- Serum aliquots will be stored in the Duke Human Vaccine Institute Accessioning Lab, the Boston at the Maxwell Finland Laboratory for Infectious Diseases, and at Cincinnati Children's Hospital until planned HAI analyses at which point the samples stored at BMC and Cincinnati will be shipped to Duke where all HAI analyses will be done

6 LABORATORY ANALYSES

6.1 Influenza Hemagglutination Inhibition (HAI) Assay

Influenza Hemagglutination Inhibition (HAI) Assays will be performed on sera collected, contingent on additional funding. Briefly, reference wild-type, reassortant, or vaccine virus strains representative of the specific viral antigens included in the 2017-2018 and 2018-2019 influenza vaccine will be used to evaluate the relative levels of all three influenza strain-specific antibodies in participant serum samples collected pre- and 28 days post-vaccination from all study participants and at 181 days post-vaccination in a subset of 100 participants at Duke. To accomplish these activities all participant samples will be interrogated for influenza antibodies against the strains of interest using the influenza hemagglutination inhibition assay (HI). This assay is considered the "gold-standard" measure by which to evaluate seroconversion/seroprotection in response to seasonal influenza vaccination. This assay will be performed in accordance with the Duke Regional Biocontainment Laboratory Virology Unit's fully optimized and approved SOP (RVUSOP004 Influenza HI of Serum Samples). Briefly, test samples will be assayed by HAI as duplicate 2-fold dilution series starting at 1:10. Serum dilutions are then incubated with a concentration of virus verified to possess a known potential for red blood cell (RBC) agglutination. The presence of virus-specific antibodies is visualized via incubation of the virus-serum mixture with a RBC solution; the endpoint titer for a given dilution series is then expressed as the reciprocal of the final dilution in which complete HAI is observed. By convention, seronegative samples are defined as having an endpoint HAI titer < 40 and seropositive samples as having an endpoint titer of ≥1:40; and seroconversion as a 4-fold change in endpoint titer relative to pre-immunization baseline or a change from <10 to ≥1:40 [37].

7 STATISTICAL CONSIDERATIONS

In collaboration with the Boston Medical Center and Cincinnati sites, the research team at Duke will oversee the statistical analysis. Data will reside on a secure Duke server maintained by Duke Health Technology Solutions (DHTS). For the study, a database will be developed and a data set for the study without personal identifiers will be made available to the CDC upon request. Duke statisticians will develop a comprehensive Statistical Analysis Plan. The summary points of the analysis plan are presented below.

7.1 Analysis Plan

Should an interim safety analysis be required, the alpha level will be adjusted to assure the overall type I error is maintained at the one-sided alpha 0.025 level for the primary outcome of non-inferiority.

7.1.1 Sample Size

Safety: Based on data from prelicensure studies [4,5,7], we assume that 5% of older adults have moderate/severe injection-site reactions after aIIV3 or IIV-HD. We have selected a clinically meaningful non-inferiority margin of 5%. Statistical calculations, without consideration of drop-out, show that with an alpha of 0.025 (one-sided), we would need 668 total subjects (334 subjects in each group across all study sites) to have at least 80% power be able to demonstrate that the proportion of moderate/severe pain was non-inferior after aIIV3 vs. IIV3-HD. Enrollment in this study shall occur during two influenza seasons (2017-18 and 2018-19).

Immunogenicity: Based on data from prelicensure studies [4,5,7], we conservatively estimate that 50% of older adults demonstrate seroconversion after vaccination with aIIV3 or IIV3-HD for the H3N2 strain. We have selected a clinically meaningful non-inferiority margin of 10%. Statistical calculations, without consider of drop-out, show that with an alpha of 0.025 (one-sided), we would need 780 total subjects (390 subjects in each group across all study sites) to have approximately 80% power to able to demonstrate that the seroconversion rate after aIIV3 was non-inferior to IIV3-HD.

The total number of subjects needed for this study was derived based on 4 factors: 1) Sample size needed for adequate statistical power to test the safety hypothesis; 2) sample size needed for adequate statistical power to test the immunogenicity hypothesis; 3) potential for subject drop-out; and 4) feasibility of dividing subjects across 2 sites. Based on these considerations, the study aims to enroll at least 880 subjects to assess both the safety and immunogenicity endpoints. The study aims to enroll at least 720 to test only the safety hypothesis.

7.1.2 Analysis Populations

Full Analysis Population:

- For Primary Objective 1 and Secondary Objective 1, the primary analysis population will be the Full Analysis Population; defined as all subjects who are randomized, vaccinated, and provide at least one day of complete data on the symptom diary.
- For Primary Objective 2 and Secondary Objective 2, the primary analysis population will be the Full Analysis Population; defined as all subjects who are randomized and vaccinated.

Immunogenicity Population:
For Primary Objective 3 and Secondary Objective 3 the primary analysis will be for the Immunogenicity Population; defined as subjects who received vaccine, provide baseline and Visit 4 blood draws of acceptable volume and quality within the protocol-defined time frame with no protocol violations affecting immunogenicity. Protocol violations affecting the immunogenicity analyses will be defined in the Statistical Analysis Plan (SAP).

The Full Analysis Population is the primary population for analysis unless otherwise stated.

7.1.3 Primary Objective 1

- To compare the proportions of moderate/severe injection site pain after aIIV3 and IIV3-HD in the full study population
  - Hypothesis: the proportion of subjects who have moderate/severe injection site pain within the first week post-vaccination will be non-inferior for allV3 compared to IIV3-HD in the full study population

This objective will be assessed using a one-sided non-inferiority test with the alpha level set at 0.025 and non-inferiority margin of 5%.

The null hypothesis is allV3 is <u>inferior</u> to IIV3-HD in regards to the proportion of subjects having moderate or severe injection site pain in the first week post vaccination.

Ho: allV3 - IIV3-HD ≥ 0.05 (5%)

The alternative hypothesis is allV3 is <u>non-inferior</u> to IIV3-HD in regards to the proportion of subjects having moderate or severe injection site pain in the first week post vaccination.

Ha: allV3 - IIV3-HD < 0.05 (5%)

The upper bound of the one-sided binomial confidence interval of the difference will be used to make this assessment.

7.1.4 Primary Objective 2

- To compare serious adverse events and events of clinical interest after aIIV3 and IIV3-HD in the full study population and by age-group

The proportion and 95% exact binomial confidence interval of serious adverse events and events of clinical interest, as well as the total number of events, will be presented by site, vaccine group, severity, and relatedness. Listings of the serious adverse events will also be presented. This information will also be prepared for the subset (N≥176) of subjects 80 or older.

7.1.5 Primary Objective 3

- To compare the seroconversion rate for the H3N2 influenza A strain after aIIV3 and IIV3-HD in the full study population
  - Hypothesis: the seroconversion rate for the H3N2 influenza A strain in the full study population after aIIV3 will be non-inferior to IIV-HD

This objective will be assessed using a one-sided non-inferiority test with the alpha level set at 0.025 and non-inferiority margin of 10%.

The null hypothesis is the allV3 H3N2 seroconversion rate is <u>inferior</u> to IIV3-HD seroconversion rate.

Ho: allV3 H3N2 rate - IIV3-HD H3N2 rate $\geq$ 0.1 (10%)

The alternative hypothesis is the allV3 H3N2 seroconversion rate is <u>non-inferior</u> to IIV3-HD H3N2 seroconversion rate.

Ha: allV3 H3N2 rate - IIV3-HD H3N2 rate < 0.1 (10%)

The upper bound of the one-sided binomial confidence interval of the difference will be used to make this assessment.

7.1.6 Secondary Objective 1

- To compare the proportions of local and systemic reactions after aIIV3 and IIV3-HD in the full study population and by age-group (65-79 years and ≥80 years) (other than moderate/severe injection site pain in the full study population)

The proportions in the full study population will be conducted as for Primary Objective 1 using a non-inferiority test to determine if allV3 is non-inferior to IIV3-HD with a 5% non-inferiority margin. These secondary objectives will be conducted with a one-sided alpha at the 0.01 level to adjust for multiple comparisons. It is recognized that with 12 different assessments at the alpha 0.01 level (given the events are truly independent) we are allowing an 11.4% chance of making a type I error.

The age-group assessments will be assessed using standard two-sided 95% confidence boundaries of the difference in proportions between the groups. No formal statistical testing will be implemented for the age-group comparisons.

7.1.7 Secondary Objective 2

- To describe and compare changes in health-related quality of life after aIIV3 and IIV3-HD in the full study population and by age-group

Each HRQOL instrument produces a summary score to measure health-related quality of life. The change in score from pre-vaccination (baseline, Visit 1) to post-vaccination (Day 3, Visit 2) time points will be compared within and between the two groups.

The Late-Life Disability and Function Instrument and Late-Life Disability and Function Instrument-Computer Adapted Test (Year 1 Only)

The function and disability sections scale raw scores are transformed to a 0 (worst health) to 100 (best health) scale. The changes from baseline will be assessed within vaccine group

using a paired t-test for the function and disability outcomes. If normality assumptions are not met, the testing will be performed with a Wilcoxon signed-rank test. The Mann-Whitney U test (Wilcoxon rank-sum test) will be used to compare the difference scores from baseline between the two vaccine groups for the function and disability outcomes.

EQ-5D-5L and Visual Analogue Scale (VAS)

The EQ-5D-5L responses are converted to a Utility Index that ranges from -0.109 (worst health) to 1.0 (best health) using the US specific value sets (http://www.euroqol.org/fileadmin/user_upload/Documenten/Excel/Crosswalk_5L/EQ-5D-5L_Crosswalk_Value_Sets.xls). The EQ VAS has a range of 0 (worst health) to 100 (best health). The changes from baseline will be assessed within vaccine group using a paired t-test for the index values and VAS. If normality assumptions are not met, the testing will be performed with a Wilcoxon signed-rank test. The Mann-Whitney U test (Wilcoxon rank-sum test) will be used to compare the difference scores from baseline between the two vaccine groups for the index values and VAS.

These health-related quality objectives will be conducted with a two-sided alpha at the 0.01 level to adjust for multiple comparisons. It is recognized that with 12 different assessments at the alpha 0.01 level (given the events are truly independent) we are allowing a 11.4% chance of making a type I error.

The statistical tests described above will also be performed for the subset (N=176) of subjects 80 or older. This testing will be considered exploratory with an alpha level of 0.05 with no alpha adjustment.

7.1.8 Secondary Objective 3

To compare serum hemagglutination inhibition (HAI) antibody titers after aIIV3 and IIV3-HD for each of the three influenza vaccine strains contained in the respective vaccine for that season in the full study population and by age-group (except for seroconversion for the H3N2 strain in the full study population)

Responses to each influenza antigen will be analyzed, with the exception of seroconversion for H3N2, which is analyzed in Primary Objective 1. The proportion of subjects with seroprotection (pre- and post-immunization) seroconversion (4-fold rise from baseline) in the two treatment groups will be presented along with 95% exact binomial confidence intervals. A 95% confidence interval of the difference in proportions between the treatment groups will also be presented.

The GMTs for each influenza antigen, including H3N2, and 95% confidence boundaries will be presented for both treatment groups. A 95% confidence interval of the difference in GMTs between the treatment groups will also be presented. This information will also be prepared for the subset (N≥176) of subjects 80 or older.

7.1.9 Sensitivity Analyses

For Primary Objective 1 a sensitivity analysis will be performed on the following sub-group of the Full Analysis Population: subjects who provided complete/informative symptom diary information for injection-site pain (i.e., eight days of completed pain field data on diary or graded

pain if present) and have no specified protocol violations as described in the Manual of Operations. This a supporting safety analysis.

7.1.10 Exploratory Objectives

The analysis for the exploratory objectives will be detailed in the comprehensive Statistical Analysis Plan.

7.2 Data Management

The novel Vanderbilt-designed resource developed specifically for online collection of research information, the Research Electronic Data Capture (REDCap) platform (https://projectredcap.org/), will be used to design study forms, including the reaction forms, and short customized questionnaires to collect information from study subjects. REDCap provides: 1) a streamlined process for rapidly building a database; 2) an intuitive interface for collecting data, with data validation and audit trail; 3) automated export procedures for seamless data downloads to common statistical packages; 4) branching logic, file uploading, and calculated fields; and 5) a quick and easy protocol set-up. This system will be used by Duke for data management. All electronic linkages will fulfill regulations for protection of human subjects and requirements to minimize the risk of breach of confidentiality.

All study-related documents containing protected health information, e.g. enrollment logs, case report forms, diaries (Appendix E) completed by study participants, will be maintained in secure research offices at Duke, Boston University, and Cincinnati Children’s Hospital, which are accessible to research staff only.

The study team will utilize a secure, encrypted, file transfer method for sharing study documents and data with the CDC. No personal identifiers will be included in any shared documents or datasets.

7.2.1 Research Electronic Data Capture (REDCap)

REDCap (http://project-redcap.org/), assists with the collection and management of data for diverse clinical and translational research studies. REDCap was designed around the concept of giving research teams an easy method to specify project needs and rapidly develop secure, web-based applications for collection, management and sharing of research data. REDCap accomplishes these key functions through use of a single study metadata table referenced by presentation-level operational modules. Based on this abstracted programming model, databases are developed in an efficient manner with little resource investment beyond the creation of a single data dictionary. The concept of metadata-driven application development is well established, and the critical factor for successful data collection lies in creating a simple workflow methodology allowing research teams to autonomously develop study-related metadata in an efficient manner. Both products include secure institutional data hosting and include full audit-trails in compliance with Health Insurance Portability and Accountability Act (HIPAA) security requirements. The REDCap Consortium is comprised of 647 active institutions. The REDCap currently supports 68,000 projects with over 89,000 users spanning numerous research focus areas across the consortium. The current project will use this software application for the design of electronic forms to collect information from study participants, to link the baseline data, sample collection date, and laboratory results in an automated database

family, to perform data cleaning and data quality assurance efficiently, and to design an analytical dataset for the analysis of the project data.

Data will be entered into the REDCap database by members of the study team from Duke, Boston Medical Center, and Cincinnati Children's Hospital using the paper case report forms utilized to record data collected as part of study procedures. Study investigators will be responsible for assuring that all paper records are securely stored according to the requirements of their IRBs. The study investigators will be responsible for assuring the accuracy of the data entered from the paper forms into REDCap. Only the assigned identifiers will be used in REDCap. Therefore, personal health identifiers will not appear in the REDCap database.

In order to perform data cleaning and data quality assurance efficiently, numerous built-in filters and checks for consistency of the data including range and limit checks, branching logic and pull down menus to limit choices for categorical variables to a pre-specified list will be implemented and performed automatically to minimize data entry error. The data will be randomly sampled and checked against source records on a regular basis. The data and related analytical datasets will also be stored at the lead and contributing sites with secured password-protected computers.

7.3 Role of the CDC Investigators in the Project

This study is funded by a CDC contract with Duke University and Boston University as Task Orders in the CISA Project Contract. The Duke University PI (Ken Schmader) will oversee the study in partnership with the Boston University PI (Elizabeth Barnett). Boston University has a subcontract with Cincinnati Children's Hospital (PI Elizabeth Schlaudecker). CDC staff will collaborate with both sites to develop the protocol, conduct the study, ensure the study is aligned with US Department of Health and Human Services (CDC) public health priorities, and analyze the data and disseminate the results. CDC may receive access to coded data not containing any directly identifying information.

8 HUMAN SUBJECTS

8.1 Human Subjects Involvement, Characteristics, and Design

Duke, Boston University, and Cincinnati Children's Hospital investigators will be responsible for submitting the protocol, informed consent (Appendix A), diaries (Appendix E), recruitment letters (Appendix M), flyers (Appendix N), and any written or verbally conveyed materials (Appendix O) specific to this project to their institutional review boards. CDC staff will be responsible for submitting materials to the CDC for Human Subjects review and approval.

To facilitate subject recruitment at the practices, we will request a waiver of consent and HIPAA authorization for ascertainment (identification, selection) and/or recruitment of potential subjects while recording identifiable private health information (PHI) prior to obtaining the subject's consent. This information will be obtained from review of the electronic scheduling and medical record systems in the clinics in order to determine eligibility for study enrollment. We will review only the minimum amount of information necessary to determine eligibility, i.e. date of birth, medical and surgical history, and recent laboratory test results. The PHI collected prior to

consent will be used to recruit and screen only. Use of PHI in this manner involves no more than minimal risk to subjects and no information will leave the study sites.

Requests for continuing review, when required, will be submitted at each engaged institution in accordance with institutional procedures. Protocol deviations or concerns about study integrity will be reported promptly to the overseeing IRB or CDC in accordance with institutional requirements.

8.2 Sources of Material

Medical history and immunization history will be obtained from the medical record and from patient report. Demographic information will be obtained from the medical record and patient report. Subjects will record solicited adverse reactogenicity events and any medical intervention sought on study days 1-8 on the symptom diary (Appendix E). Diary information will be reported to the study team during a telephone call. The research staff will assess one or more of the following: weight, height, temperature, blood pressure, and pulse.

8.3 Potential Risks and Benefits

aIIV3 or IIV-HD3 are FDA-licensed vaccines approved for use in adults ≥65 years old. Both vaccines are standard clinical practice and recommended by the CDC. Participants will be provided with the CDC Vaccine Information Statement (VIS) for IIV (https://www.cdc.gov/vaccines/hcp/vis/vis-statements/flu.pdf).

IIV risks include minor problems such as soreness, redness, swelling, or pain where the shot was given, hoarseness, sore, red or itchy eyes, cough, fever, aches, headache, itching, fatigue, all of which usually occur within 1-2 days of vaccination and are self-limiting. Some people get severe pain in the shoulder and have difficulty moving the arm where a shot was given. This happens very rarely. Syncope (fainting) can occur in association with administration of injectable vaccines. Sitting or lying down for about 15 minutes can help prevent fainting, and injuries caused by a fall, as recommended in the ACIP General Recommendations on Immunization[1]. Subjects should inform their doctor should they feel dizzy, or have vision changes or ringing in the ears. More serious problems including a small increased risk of Guillain-Barré Syndrome estimated at 1 or 2 additional cases per million people vaccinated. This is much lower than the risk of severe complications from influenza infection, which can be prevented by IIV[38]. In addition, any medication can cause a severe allergic reaction, or anaphylaxis, which is estimated at ~ 1 in one million doses of IIV administered[39].

Risks of blood drawing include pain, swelling, bleeding, or bruising at the site where the blood sample is collected. Subjects may also experience dizziness or fainting. There is a small risk of infection around the vein where the blood was collected. Each study subject will be asked to have up to 3 blood samplings with the total volume not to exceed 30mL over approximately 6 month period of time. Participants who return for repeat vaccination in the second year will be asked to have 2 additional blood sampling with the volume in the second year not to exceed 20 mL.

As with any licensed vaccine, protection may not occur in 100% of vaccinated persons.

An additional risk of study participation is the potential for loss of confidentiality.

8.4 Adequacy of Protection Against Risks

8.4.1 Protections against Risk

To decrease the possibility of infection at the site of blood drawing, the area on the arm above the vein where blood will be taken will be prepped with 70% isopropyl alcohol antiseptic prior to venipuncture.

Subjects will be counseled on possible side effects following vaccination and followed closely during the 8 days post-vaccination for assessment of moderate to severe local or systemic reactogenicity. Subjects will be evaluated and cared for as described in the Unscheduled Visit section above. All subjects will be monitored in a sitting or lying position for 15 minutes following vaccinations to help prevent fainting, and injuries caused by a fall. Subjects with a prior history of severe allergic reaction after a previous dose of any influenza vaccine, or to a vaccine component, including egg protein, will be excluded from study enrollment. Data Safety monitoring, as described above (Section 5.4.3 and Appendix H), shall also be done.

The study team will provide documentation to the participant and primary care provider regarding receipt of influenza vaccine without specification of whether it was high dose or adjuvanted vaccine to preserve blinding.

If a participant’s care requires the identity of the vaccine received, blinding will be broken for that patient. At the end of the study, the participants and providers will receive documentation about which vaccine the participant received.

Every effort possible will be made to keep information about participants confidential. Computerized participant information will be kept in password-protected files on secured servers. Paper case report forms will be kept in locked files belonging to the study personnel. Any publications resulting from this work will not contain any identifiable participant information.

8.4.2 *ClinicalTrials.gov Requirements*

The project is registered on ClinicalTrials.gov (NCT # NCT03183908).

8.5 Human Subjects

In obtaining and documenting informed consent, the Investigator and study team will comply with the applicable regulatory requirements, Good Clinical Practices, and ethical principles. The written informed consent form must be signed and dated by the study participant prior to initiation of any study activities.

8.5.1 Vulnerable Subjects Research

This study proposes to include subjects with mild cognitive impairment. All potential subjects will undergo cognitive assessment to ensure they are capable of providing consent. Mild cognitive impairment is a common age-related condition that is defined by the presence of short-term memory impairment that does not interfere the individual’s ability to perform activities of daily living or affect other areas of cognition, including judgment and independent decision-making. Therefore, persons with mild cognitive impairment have the capacity to make decisions about their health care choices, including influenza vaccination, and participation in research studies. Influenza vaccination is recommended for these individuals. The benefits and burdens of the proposed study apply equally to these individuals as to persons without mild cognitive impairment.

Potential participants may have low scores on the cognitive screening tests and be ineligible for the study. In this case the study doctor or designee will review the results with the individual and recommend follow-up with the individual’s health care provider.

REFERENCES

1. Grohskopf LA, Sokolow LZ, Broder KR, et al. Prevention and Control of Seasonal Influenza with Vaccines: Recommendations of the Advisory Committee on Immunization Practices - United States, 2017-18 Influenza Season. MMWR Recomm Rep 2017;66:1-20.
2. Grohskopf LA, Sokolow LZ, Broder KR, et al. Prevention and Control of Seasonal Influenza with Vaccines. MMWR Recomm Rep 2016;65:1-54.
3. Bandaranayake T, Shaw AC. Host Resistance and Immune Aging. Clin Geriatr Med 2016;32:415-32.
4. Deng Y, Jing Y, Campbell AE, Gravenstein S. Age-related impaired type 1 T cell responses to influenza: reduced activation ex vivo, decreased expansion in CTL culture in vitro, and blunted response to influenza vaccination in vivo in the elderly. J Immunol 2004;172:3437-46.
5. Shahid Z, Kleppinger A, Gentleman B, Falsey AR, McElhaney JE. Clinical and immunologic predictors of influenza illness among vaccinated older adults. Vaccine 2010;28:6145-51.
6. Smeeth L, Thomas SL, Hall AJ, Hubbard R, Farrington P, Vallance P. Risk of myocardial infarction and stroke after acute infection or vaccination. N Engl J Med 2004;351:2611-8.
7. Thompson WW, Shay DK, Weintraub E, et al. Mortality associated with influenza and respiratory syncytial virus in the United States. JAMA 2003;289:179-86.
8. Centers for Disease C, Prevention. Estimates of deaths associated with seasonal influenza --- United States, 1976-2007. MMWR Morb Mortal Wkly Rep 2010;59:1057-62.
9. Zhou H, Thompson WW, Viboud CG, et al. Hospitalizations associated with influenza and respiratory syncytial virus in the United States, 1993-2008. Clin Infect Dis 2012;54:1427-36.
10. Barker WH, Borisute H, Cox C. A study of the impact of influenza on the functional status of frail older people. Arch Intern Med 1998;158:645-50.
11. Jefferson T, Di Pietrantonj C, Al-Ansary LA, Ferroni E, Thorning S, Thomas RE. Vaccines for preventing influenza in the elderly. Cochrane Database Syst Rev 2010:CD004876.
12. Keitel WA, Atmar RL, Cate TR, et al. Safety of high doses of influenza vaccine and effect on antibody responses in elderly persons. Arch Intern Med 2006;166:1121-7.
13. Fluzone High-Dose Package Insert. 2016. at http://www.fda.gov/downloads/BiologicsBloodVaccines/Vaccines/ApprovedProducts/UCM305079.pdf.)
14. DiazGranados CA, Dunning AJ, Kimmel M, et al. Efficacy of high-dose versus standard-dose influenza vaccine in older adults. N Engl J Med 2014;371:635-45.
15. Falsey AR, Treanor JJ, Tornieporth N, Capellan J, Gorse GJ. Randomized, double-blind controlled phase 3 trial comparing the immunogenicity of high-dose and standard-dose influenza vaccine in adults 65 years of age and older. J Infect Dis 2009;200:172-80.
16. Tsai TF. Fluad(R)-MF59(R)-Adjuvanted Influenza Vaccine in Older Adults. Infect Chemother 2013;45:159-74.
17. FDA approves first seasonal influenza vaccine containing an adjuvant. 2015. at http://www.fda.gov/NewsEvents/Newsroom/PressAnnouncements/ucm474295.htm.)
18. FLUAD US Package Insert. 2016. at http://www.fda.gov/downloads/BiologicsBloodVaccines/SafetyAvailability/VaccineSafety/UCM474387.pdf.)
19. Katz S, Ford AB, Moskowitz RW, Jackson BA, Jaffe MW. Studies of Illness in the Aged. The Index of Adl: A Standardized Measure of Biological and Psychosocial Function. JAMA 1963;185:914-9.

20. Lawton MP, Brody EM. Assessment of older people: self-maintaining and instrumental activities of daily living. Gerontologist 1969;9:179-86.
21. Phelan EA, Anderson LA, LaCroix AZ, Larson EB. Older adults' views of "successful aging"--how do they compare with researchers' definitions? J Am Geriatr Soc 2004;52:211-6.
22. Borson S, Scanlan JM, Chen P, Ganguli M. The Mini-Cog as a screen for dementia: validation in a population-based sample. J Am Geriatr Soc 2003;51:1451-4.
23. Herr KA, Mobily PR, Kohout FJ, Wagenaar D. Evaluation of the Faces Pain Scale for use with the elderly. Clin J Pain 1998;14:29-38.
24. Bradt J, Dileo C, Magill L, Teague A. Music interventions for improving psychological and physical outcomes in cancer patients. Cochrane Database Syst Rev 2016:CD006911.
25. Haley SM, Jette AM, Coster WJ, et al. Late Life Function and Disability Instrument: II. Development and evaluation of the function component. J Gerontol A Biol Sci Med Sci 2002;57:M217-22.
26. Hand C, Richardson J, Letts L, Stratford P. Construct validity of the late life function and disability instrument for adults with chronic conditions. Disabil Rehabil 2010;32:50-6.
27. Jette AM, Haley SM, Coster WJ, et al. Late life function and disability instrument: I. Development and evaluation of the disability component. J Gerontol A Biol Sci Med Sci 2002;57:M209-16.
28. McDonough CM, Tian F, Ni P, et al. Development of the computer-adaptive version of the Late-Life Function and Disability Instrument. J Gerontol A Biol Sci Med Sci 2012;67:1427-38.
29. Brazier J, Jones N, Kind P. Testing the validity of the Euroqol and comparing it with the SF-36 health survey questionnaire. Qual Life Res 1993;2:169-80.
30. Herdman M, Gudex C, Lloyd A, et al. Development and preliminary testing of the new five-level version of EQ-5D (EQ-5D-5L). Qual Life Res 2011;20:1727-36.
31. van Hout B, Janssen MF, Feng YS, et al. Interim scoring for the EQ-5D-5L: mapping the EQ-5D-5L to EQ-5D-3L value sets. Value Health 2012;15:708-15.
32. Gandek B, Ware JE, Aaronson NK, et al. Cross-validation of item selection and scoring for the SF-12 Health Survey in nine countries: results from the IQOLA Project. International Quality of Life Assessment. J Clin Epidemiol 1998;51:1171-8.
33. Ware J, Jr., Kosinski M, Keller SD. A 12-Item Short-Form Health Survey: construction of scales and preliminary tests of reliability and validity. Med Care 1996;34:220-33.
34. Coplan PM, Schmader K, Nikas A, et al. Development of a measure of the burden of pain due to herpes zoster and postherpetic neuralgia for prevention trials: adaptation of the brief pain inventory. J Pain 2004;5:344-56.
35. Schmader KE, Sloane R, Pieper C, et al. The impact of acute herpes zoster pain and discomfort on functional status and quality of life in older adults. Clin J Pain 2007;23:490-6.
36. Schmader KE, Johnson GR, Saddier P, et al. Effect of a zoster vaccine on herpes zoster-related interference with functional status and health-related quality-of-life measures in older adults. J Am Geriatr Soc 2010;58:1634-41.
37. Katz JM, Hancock K, Xu X. Serologic assays for influenza surveillance, diagnosis and vaccine evaluation. Expert Rev Anti Infect Ther 2011;9:669-83.
38. Kwong JC, Vasa PP, Campitelli MA, et al. Risk of Guillain-Barre syndrome after seasonal influenza vaccination and influenza health-care encounters: a self-controlled study. Lancet Infect Dis 2013;13:769-76.
39. McNeil MM, Weintraub ES, Duffy J, et al. Risk of anaphylaxis after vaccination in children and adults. J Allergy Clin Immunol 2016;137:868-78.

APPENDIX

Appendix A: Written Informed Consent
Appendix B: Mini-Cog
Appendix C: Rowland Universal Dementia Assessment Scale (RUDAS)
Appendix D: Treatment Administration Record/Immediate Reactogenicity Assessment Form
Appendix E: Patient Symptom Diary
Appendix F: FACES Pain Scale
Appendix G: Immune-Mediated Conditions
Appendix H: Safety Monitoring Plan
Appendix I: LLFDI-CAT
Appendix J: EQ-5D-5L
Appendix K: Vaccine Reaction Questionnaire
Appendix L: Perceptions of the Vaccination Experience
Appendix M: Recruitment Letter
Appendix N: Flyer
Appendix O: Phone Script

Appendix A: Written Informed Consent

Appendix B: Mini-Cog

Appendix C: Rowland Universal Dementia Assessment Scale (RUDAS)

Appendix D: Treatment Administration Record/Immediate Reactogenicity Assessment Form

Appendix E: Patient Symptom Diary

Appendix F: FACES Pain Scale

Appendix G: Immune-Mediated Conditions

**Gastrointestinal disorders**	**Liver disorders**
• Celiac disease<br>• Crohn’s disease<br>• Ulcerative colitis<br>• Ulcerative proctitis	• Autoimmune cholangitis<br>• Autoimmune hepatitis<br>• Primary biliary cirrhosis<br>• Primary sclerosing cholangitis
**Musculoskeletal disorders**	**Neuroinflammatory disorders**
• Antisynthetase syndrome<br>• Dermatomyositis<br>• Mixed connective tissue disorder<br>• Polymyalgia rheumatic<br>• Polymyositis<br>• Psoriatic arthropathy<br>• Relapsing polychondritis<br>• Rheumatoid arthritis<br>• Scleroderma, including diffuse systemic form and CREST syndrome<br>• Spondyloarthritis, including ankylosing spondylitis, reactive arthritis (Reiter's Syndrome) and<br>undifferentiated spondyloarthritis<br>• Systemic lupus erythematosus<br>• Systemic sclerosis	• Acute disseminated encephalomyelitis, including site specific variants (e.g., non-infectious<br>encephalitis, encephalomyelitis, myelitis, myeloradiculomyelitis)<br>• Cranial nerve disorders, including paralyses/paresis (e.g., Bell’s palsy)<br>• Guillain-Barré syndrome, including Miller Fisher syndrome and other variants<br>• Immune-mediated peripheral neuropathies and plexopathies, including chronic inflammatory demyelinating polyneuropathy, multifocal motor neuropathy and polyneuropathies associated with monoclonal gammopathy<br>• Multiple sclerosis<br>• Narcolepsy<br>• Optic neuritis<br>• Transverse myelitis<br>• Myasthenia gravis, including Eaton-Lambert syndrome
**Metabolic diseases**	**Skin disorders**
• Addison’s disease<br>• Autoimmune thyroiditis (including Hashimoto thyroiditis)<br>• Diabetes mellitus type I<br>• Grave's or Basedow’s disease	• Alopecia areata<br>• Autoimmune bullous skin diseases, including pemphigus, pemphigoid and dermatitis<br>herpetiformis<br>• Cutaneous lupus erythematosus<br>• Erythema nodosum<br>• Erythema multiforme<br>• Morphoea<br>• Lichen planus<br>• Psoriasis<br>• Sweet’s syndrome<br>• Vitiligo
**Vasculitides**	**Others**
• Large vessels vasculitis including: giant cell arteritis such as Takayasu's arteritis and temporal<br>arteritis	• Antiphospholipid syndrome<br>• Autoimmune hemolytic anemia<br>• Autoimmune glomerulonephritis (including IgA nephropathy, glomerulonephritis rapidly

• Medium sized and/or small vessels vasculitis including: polyarteritis nodosa, Kawasaki's disease, microscopic polyangiitis, Wegener's granulomatosis, Churg–Strauss syndrome (allergic granulomatous angiitis), Buerger’s disease thromboangiitis obliterans, necrotizing vasculitis and anti-neutrophil cytoplasmic antibody (ANCA) positive vasculitis (type unspecified), Henoch-Schonlein purpura, Behcet's syndrome, leukocytoclastic vasculitis	progressive, membranous glomerulonephritis, membranoproliferative glomerulonephritis, and mesangioproliferative glomerulonephritis)<br>• Autoimmune myocarditis/cardiomyopathy<br>• Autoimmune thrombocytopenia<br>• Goodpasture syndrome<br>• Idiopathic pulmonary fibrosis<br>• Pernicious anemia<br>• Raynaud’s phenomenon<br>• Sarcoidosis<br>• Sjögren’s syndrome<br>• Stevens-Johnson syndrome<br>• Uveitis

Appendix H: Safety Monitoring Plan

The safety monitoring plan includes 3 types of adverse events described in the Table below. The plan includes two components: an ongoing adverse event case report review, and an interim safety data review with the expert safety panel.

Type A adverse events	Type B adverse events	Type C adverse events
o Syncope occurring post-vaccination while patient is under clinic observation for Day 1 study activities.<br>o Anaphylaxis within 24 hours of vaccination<br>o Guillain-Barré syndrome (GBS) occurring within 42 days of vaccination<br>o Death within 42 days following vaccination	o Serious adverse events (SAE) occurring within 42 days of vaccination, unless unrelated to vaccine<br>o New onset immune-mediated diseases occurring within 42 days of vaccination other than GBS (Appendix G), unless unrelated to vaccine	o Severe injection-site reactions occurring within 7 days of vaccination (Table 2)<br>o Severe fever occurring within 7 days of vaccination (Table 3)

Ongoing AE case report review will be performed as follows:

- A site identifying a Type A AE in a study subject should notify study investigators at both sites and CDC within one business day of identifying the AE. Site investigators should make an assessment as to relatedness to study product (related, possibly related, unlikely related or unrelated) and provide this information when available, but should not delay reporting.

- A site identifying a Type B AE should make an assessment as to relatedness to study product before reporting it. An SAE that is considered to be related or possibly related to vaccine should be reported to study investigators at both sites and CDC within one business day of making this relatedness determination. Type B AEs that are not related do not need to be urgently reported.

- Type C AEs do not need to be reported as part of ongoing monitoring, unless they also result in an SAE.

Information about AE case reports that is shared with the study teams will be blinded with respect to vaccine product used. Reporting to the respective IRB will be per the respective institutional IRB requirements and will also initially be blinded. Sites will provide narrative case reports for Type A AEs and Type B AEs (unless unrelated to vaccine) to CDC within 1 week. No personal identifiable information (PII), including date of vaccination, should be included. Discussions about AE case reports for Type A and B events will take place on routine or ad hoc calls with the study investigators and team. If a site or CDC have clinical safety concerns that they feel need more urgent attention, then the study team will meet promptly to determine a plan of action, which might include consultation with the safety monitoring panel. The following events should lead to a prompt study team meeting:

- If there are 3 or more Type A syncope events
- If there are 2 or more other Type A AEs

- If there are 2 or more other serious AEs or new onset autoimmune diseases (unless unrelated to the study vaccine) that are clinically similar (e.g. same organ class)
- If a clinical investigator involved with the study has a safety concern not specified above that he/she feels needs prompt attention.

The interim safety data review will provide an opportunity to make changes to the protocol or consent before the second year of influenza study enrollment. This data review will include either the first 200 subjects vaccinated with the opportunity for 42 days of follow-up OR all subjects vaccinated with 42 days of follow at the time the report is prepared. This review will include all randomized subjects who received vaccine, including those with protocol violations or who dropped out or died. The review will maintain blinding and will describe the following in aggregate and will also be stratified into two age groups: 65-79 and ≥80 years.

- Proportion of each Type A AE (all, related, possibly related, unlikely related or unrelated)
- Proportion of serious AEs (including any Type A AEs also considered serious) (all, related, possibly related, unlikely related, or not related)
- Proportion new immune-mediated diseases (except GBS) (all, related, possibly related, unlikely related, or not related) (Appendix G)
- Proportion of each severe injection-site reaction (Table 2) and severe fever (assume all related)

The interim review will include clinical summary line lists for the AEs described above, except for the reactogenicity events. An appendix of clinical narratives for all Type A and Type B AEs will also be included. The Duke statistical team in collaboration with the study team will prepare and present this summary. Background information about reactogenicity frequencies from the package inserts will be provided. This information will be shared and reviewed with the expert safety panel. The safety panel will consider any concerning findings and determine if there is need to consider additional actions including statistical analyses and reassessment, changes to study eligibility or procedures to minimize or assess risk, or addition of information to the informed consent. The safety panelists will provide individual expert input but will not make final decisions. There are no pre-specified statistical criteria to guide decisions; rather clinical judgement will be used. For all of these steps study investigators and the safety panel will see the same data. The safety panel may make any of the following suggestions at the end of the interim data review.

- No substantial safety concerns and either:
    - No further analysis and continue routine safety monitoring
    - There are some small changes recommended in routine monitoring such as adding a new AE.
- Specific safety concern:
    - If there is a specific safety concern, such as a higher than expected proportion of severe fever after vaccine, than the first step will be to clinically describe the AEs without breaking randomization or blinding. For example, the duration of the fever could be assessed and the level of medical utilization.
    - If after in-depth clinical review, a concern remains, then the next step will be to break randomization into treatment groups (while maintaining blinding). If there is an isolated concern, then it may be sufficient to do this only for the specific AE. For example, if there are 5 SAEs of kidney failure and after breaking into two groups, no imbalance is seen, that may be sufficient.

    - If it is clinically needed, then blinding may be broken either for a selected AE or for the full interim data.
- Substantial safety concerns:
    - The interim safety dataset will be analyzed by vaccination group and blinding will be discontinued for these data.

The safety panel will consist of three to four expert physicians who are not investigators on this study. The physicians will consist of staff from the CDC and from CISA site(s) other than Duke or BMC, including at least one internal medicine physician. The panel will meet initially before study enrollment to review the study and the monitoring plan and to share background information about what is known about aIIV and IIV3-HD safety (package insert review). A meeting will be scheduled in 2018 for an interim review of safety data. Study investigators and statisticians will be invited to participate, and the meetings will consist of open discussions between the panel and study investigators (under CISA confidentially agreement). Ad hoc meetings will be scheduled as needed.

Appendix I: LLFDI-CAT

Appendix J: EQ-5D-5L and VAS

Appendix K: Vaccine Reaction Questionnaire

Appendix L: Perceptions of the Vaccination Experience

Appendix M: Recruitment Letter

Dear Sir or Madam,

The Duke Division of Geriatrics and the Duke Vaccine and Trials Unit are conducting an influenza vaccine research study. We are contacting you because as an older adult you or someone you know may be interested in this study. Every eligible study participant will be randomized (like flipping a coin), to receive an injection of either FLUAD™, an adjuvanted inactivated influenza vaccine, or Fluzone® High-Dose, inactivated influenza vaccine. An adjuvant is a substance added to a vaccine to increase the immune response. Both vaccines are currently licensed and approved in the United States for older people.

Vaccines can help prevent infection and disease. Vaccines work by causing the body to make proteins called antibodies that fight infection. When you get flu vaccine (sometimes called a flu shot), your immune system makes antibodies against the flu virus.

The **purpose** of this research study is to determine if there is a difference in older people for side effects following vaccination, and also to compare the immune response to the two flu vaccines. Previous studies have shown that both vaccines provide protection from the flu in older people.

<u>What is involved with this study?</u>

- Come in for **2 visits**: baseline and day twenty-nine. You will receive the vaccine at the baseline visit
- Have about **2 teaspoons** of blood taken from your arm at each visit
- Have your temperature, blood pressure and pulse measured at each visit
- Complete post vaccination assessments and questionnaires
- Receive **3 telephone calls** following your vaccination to review questionnaires
- Receive payment for your time and travel

If you would like to participate, please call the study team at 919-660-7581 or 919-668-8728.

Sincerely,

Kenneth Schmader, M.D.,
Principal Investigator
Chief, Division of Geriatrics
Department of Medicine

Pro00083845

Appendix N: Flyer

Appendix O: Phone Script

Hi, this is ______________________________calling from the Division of Geriatrics/Duke Clinical Vaccine Unit. Thank you for your interest in this influenza vaccine research study.
I would like to tell you about this study and then, if you are still interested, ask you a few questions about your medical history to see if you qualify for participation.

Are you still interested in learning more about this study? **☐Yes** **☐No**

If no, thank them for their time.

Principal Investigator and Purpose

The principal investigator is Dr. Kenneth Schmader. The purpose of this study is to compare the safety of FLUAD™ and Fluzone® High-Dose in older people and to see if one vaccine or the other helps older adults make protective antibodies better against the flu.

Procedures

As part of this study, you will be asked to come in for 2 study visits and you will receive 3 scheduled phone calls over a 6-week period.
Visit 1 (clinic visit) Study Day #1: Study staff will explain the study, review the consent form with you, and answer any questions you may have. You will then read and sign the consent form. Your medical history, medications that you are currently taking, as well as study criteria will be reviewed, to make sure that you qualify for the study. Study staff will take your temperature, blood pressure, and heart rate. You will be asked about your history of flu vaccination, and will you be asked about any pain, swelling, or redness you are experiencing in your arm before vaccination. You will also be asked questions about your quality of life. A blood sample of 10 mL (2 teaspoons) will be taken to test for antibody levels. You will be randomly assigned (like flipping a coin) to receive either the FLUAD™ or Fluzone® High-Dose flu vaccine. You will need to stay in the clinic for at least 15 minutes after that to be watched for any reactions. You will be given a symptom diary form, measurement tool and thermometer, and shown how to use them for the study.
Visit 2 (phone call follow-up) Study Day #3: A member of the study staff will contact you to review the information about your symptoms and medications that you should have been recording daily in the symptom diary. You will also be asked about your quality of life.
Visit 3 (phone call follow-up) Study Day #9: A member of the study staff will contact you again to review the information about your symptoms and medications that you should have been recording daily in the symptom diary. You will also be asked about your quality of life, and about your experience with this study.
Visit 4 (clinic visit) Study Day #29: About 28 days after the first visit you will return to the clinic for another in-person visit. You will be asked about your health and any medicines you are taking. A blood sample of 10 mL (2 teaspoons) will be taken to test for antibody levels.

<u>Visit 5 (phone call follow-up) Study Day #43:</u> A member of the study staff will contact you to ask you about your symptoms and medications since your last visit.

<u>Risks</u>

Possible risks with receiving the Flu vaccine include: redness, swelling, or pain where the shot was given; fever, body aches, headache, or fatigue; nausea; cough or hoarseness; sore, red or itchy eyes; or itching.
Guillain-Barré syndrome (GBS) is a rare but serious condition that can occur after certain infections or after receiving certain vaccines such as the flu vaccine. There is a small increased risk of GBS (about 1 or 2 additional cases per million people vaccinated) after vaccination with flu vaccine. GBS causes inflammation and damage to the nerves in your body. Minor symptoms such as muscle tiredness or more severe symptoms, such as paralysis (weakness, or inability to move certain parts of the body) may occur.

<u>Benefits/Compensation</u>

There may be direct medical benefit to you. Study participation will confirm that you receive the recommended flu vaccination. The two flu vaccines in this study have both been shown to prevent influenza in older people. You may develop protective antibodies against influenza. Information learned from this study may also help researchers understand if there are difference in how older people respond to FLUAD™ and Fluzone® High-Dose vaccines.
All study participants will be compensated $50 after completing each blood draw visit and $25 after completing each phone contact.

<u>Consent for Prescreening/Confidentiality</u>

I will need to ask a few questions about your medical history to determine if you potentially qualify to take part in this study. Answering is voluntary, and refusing to answer will have no effect on your status as a patient, employee or volunteer at Duke, but we will not be able to schedule you if you choose not to answer the questions.

The information we collect is kept confidential, and if, for any reason, you do not qualify for this study, none of your answers to any questions will be kept.

May I ask these questions? **☐Yes** **☐No**
If no, thank them for their time.

STUDY CANDIDATE QUESTIONNAIRE

What is your age? _____ **(Must be ≥ 65)**

Have you already received your flu shot for the current flu season? □Yes □No
(Answering "YES" is exclusionary)

Have you been a participant in a research study involving an investigational product (drug/biologic/device) within the past 30 days?
Co-enrollment in observational or behavioral intervention studies are allowed at any time, enrollment in a clinical trial involving and investigational product (other than vaccine) may occur after 30 days following vaccine receipt. □Yes □No
(Answering "YES" is exclusionary)

Do you have confirmed or suspected immunosuppressive condition resulting from disease (Malignancy, HIV), or currently undergoing treatment for cancer (chemotherapy or radiation therapy) within the preceding 12 months? □Yes □No
(Answering "YES" is exclusionary)

Do you have an active neoplastic disease (excluding non-melanoma skin cancer or prostate cancer that is stable in the absence of therapy) or a history of any hematologic malignancy?
(Participants with a history of malignancy may be included if, after previous treatment by surgical excision, chemotherapy or radiation therapy, the participant has been observed for a period that in the investigator's estimation provides a reasonable assurance of sustained cure.)
(Answering "YES" is exclusionary) □Yes □No

Do you have a history of thrombocytopenia (low platelet count), bleeding disorders or anticoagulant use that may interfere with intramuscular injection? □Yes □No
(Answering "YES" is exclusionary)

Have you received any blood transfusions in the past 3 months?
(Answering "YES" is exclusionary) □Yes □No

Have you received any vaccinations in the past 6 weeks or planned vaccinations during the 42 day post vaccination period (including pneumococcal vaccines)?
(Receipt of Shingrix or HEPLISAV-B within 6 weeks prior to enrollment in this study, or planning receipt of Shingrix during the 42 days post vaccination is exclusionary) □Yes □No
(Answering "YES" is exclusionary)

Do you currently have an acute illness with a fever of 100.0°F? □Yes □No
(Answering "YES" is exclusionary)

Have you had a severe allergic reaction after a previous dose of any influenza vaccine a vaccine component (Formaldehyde, Octylphenol ethoxylate, neomycin, kanamycin, barium, or cetyltrimethylammonium bromide (CTAB)), allergy to egg proteins, or latex, or history of Guillain-Barré syndrome? □Yes □No
(Answering "YES" is exclusionary)

Do you have a history of substance abuse? □Yes □No
(Answering "YES" is exclusionary)

What medications (including medication dose) and supplements are currently taking?
____
____
____
____
____
____

Do you have a history of any of the following medical conditions?

Liver disease	___Yes	___ No
Cognitive Impairment	___Yes	___ No
Endocrine disease	___Yes	___ No
Kidney disease	___Yes	___ No
Gastrointestinal disease	___Yes	___ No
Cardiovascular disease	___Yes	___ No
HIV or Hepatitis A, B or C	___Yes	___ No
Coronary Artery disease	___Yes	___ No
Cancer	___Yes	___ No
Pulmonary disease	___Yes	___ No

If yes to any of the above, describe:____
____
____
____
(Any condition that, in the investigator's opinion, might pose a health risk to the candidate or adversely affect compliance is exclusionary)

(If candidate is eligible, please provide the following information)

Name ___________________________________

Address ___________________________________

____
____

e-mail address:______________________________

Phone #: Primary ______________________ Type _____
Secondary____________________ Type _____

Gender: Male _____ Female ____ Date of birth: _____/_____/_____

Screening date & time: _____/_____/_____ @ _____:______ (24 hour)
mm dd yy

Duke patient? Yes ____ No ____ MRN, if applicable ___________________

Finish with this Maestro Scheduling statement

I need to ask your permission to use your health information to schedule your initial study visit in Duke's electronic scheduling system prior to your visit. If you agree to grant your permission to use your health information in this way, it means that your information will be linked to this study and may be seen by members of the study team and the Duke personnel who are associated with scheduling. The only risk to you in agreeing to this use of your information is the risk of loss of confidentially. However, all of the individuals who will see your information and its link to the study are trained professionals who work with healthcare information daily and are aware of the importance of maintaining confidentiality of health records. Your health information will be used solely to pre-schedule your initial visit prior to the formal written consent process.

May I have your permission to use your health information to schedule this research visit, with the understanding that this in no way obligates you to participate in the study?

Yes_____ No_____ Date_____/_____/_____ Time____:_____ **(24 hour)**
mm dd yy

___________________________ _____________________
Interviewer's signature **Date**
