# Supplementary material for: Safety, Reactogenicity, and Health-Related Quality of Life After Trivalent Adjuvanted vs Trivalent High-Dose Inactivated Influenza Vaccines in Older Adults: A Randomized Clinical Trial
Source: JAMA Netw Open. 2021 Jan 14;4(1):e2031266. doi: 10.1001/jamanetworkopen.2020.31266 (PMC7809592; doi:10.1001/jamanetworkopen.2020.31266)
Supplement: Supplement 2. — eAppendix 1. Eligibility Criteria eAppendix 2. Reactogenicity Assessment eTable. Local and Systemic Reactions during Days 1 through 8 Following Trivalent Adjuvanted Inactivated Influenza Vaccine (aIIV3) and Trivalent High-Dose Inactivated Influenza Vaccine (HD-IIV3) by Age Group in Older Adults [file jamanetwopen-e2031266-s002.pdf]

Supplemental Online Content

Schmader KE, Liu CK, Harrington T, et al. Safety, reactogenicity, and health-related quality of life after trivalent adjuvanted vs trivalent high-dose inactivated influenza vaccines in older adults: a randomized clinical trial. *JAMA Netw Open*. 2021;4(1):e2031266. doi:10.1001/jamanetworkopen.2020.31266

eAppendix 1. Eligibility Criteria
eAppendix 2. Reactogenicity Assessment
eTable. Local and Systemic Reactions during Days 1 through 8 Following Trivalent Adjuvanted Inactivated Influenza Vaccine (aIIV3) and Trivalent High-Dose Inactivated Influenza Vaccine (HD-IIV3) by Age Group in Older Adults

This supplemental material has been provided by the authors to give readers additional information about their work.

© 2021 Schmader KE et al. *JAMA Network Open.*

eAppendix 1. Eligibility Criteria

Subject Inclusion Criteria

1. Persons aged ≥65 years, living in the community
2. Intention of receiving IIV vaccine based on ACIP-CDC guidelines
3. Willing to provide written informed consent prior to initiation of any study procedures
4. Able to speak English
5. Able and willing to complete baseline assessments and questionnaires, and to allow information to be collected from their electronic medical record
6. Able and willing to complete post-vaccine assessments and questionnaires independently or with assistance
7. Able and willing to have blood drawn for the study
8. Able and willing to return in about one month for a follow-up visit including completing questionnaires and having another blood test
9. Access to and ability to use a phone, independently or with assistance
10. Adequate vision and motor skills to complete the symptom diary form independently or with assistance.
11. Not living in a skilled nursing facility/nursing home/long term acute care facility

Subject Exclusion Criteria

1. Influenza vaccine receipt during the current influenza season prior to study enrollment
2. Enrolled in this study during the 2017-18 (Year 1) influenza season
   Note: Year 1 study participant will only be enrolled in Year 2 if they are participating in the sub-study on repeat vaccination
3. Has immunosuppression as a result of an underlying illness or treatment, or use of anti-cancer chemotherapy or radiation therapy within the preceding 12 months.
4. Has an active neoplastic disease (excluding non-melanoma skin cancer or prostate cancer that is stable in the absence of therapy) or a history of any hematologic malignancy*
   **Participants with a history of malignancy may be included if, after previous treatment by surgical excision, chemotherapy or radiation therapy, the participant has been observed for a period that in the investigator's estimation provides a reasonable assurance of sustained cure*
5. Thrombocytopenia, bleeding disorder, or anticoagulant use contraindicating intramuscular injection
6. Receipt of blood or blood-derived products in the past three months
7. History of febrile illness (≥ 100.0°F or 37.8°C) within the past 24 hours prior to IIV administration (temporary deferral)
8. Contraindication to IIV receipt including history of severe allergic reaction after a previous dose of any influenza vaccine; or to a vaccine component*, including egg protein; or a latex allergy
   **Formaldehyde, Octylphenol ethoxylate, neomycin, kanamycin, barium, cetyltrimethlyammonium bromide (CTAB)*
9. Any history of Guillain-Barré syndrome
10. Mild to severe dementia as determined by the Mini-Cog tool and the Rowland Universal Dementia Assessment Scale (RUDAS)
11. Substance use that could interfere with study compliance

© 2021 Schmader KE et al. *JAMA Network Open.*

12. Receipt of any inactivated licensed vaccine within 2 weeks, or live attenuated licensed vaccine within 4 weeks prior to enrollment in this study, or planning receipt of any vaccines during the 42 days post-vaccination period (including pneumococcal vaccines)
13. Receipt of Shingrix (Zoster Vaccine Recombinant, Adjuvanted) or HEPLISAV-B (Hepatitis B Vaccine (Recombinant), Adjuvanted) vaccine within 6 weeks prior to enrollment in this study, or planning receipt of Shingrix or HEPLISAV-B during the 42 days post-vaccination period.
14. Anyone who is already enrolled or plans to enroll in another clinical trial with an investigational product within 28 days of vaccine receipt. Co-enrollment in observational or behavioral intervention studies are allowed at any time while enrollment in a clinical trial involving an investigational product (other than vaccine) may occur after 30 days following vaccine receipt.
15. Hearing loss determined by the investigators to prevent successful communication over the phone
16. Any condition which, in the opinion of the investigators, may pose a health risk to the subject or interfere with the evaluation of the study objectives.
17. Anyone who is a relative or subordinate of any research study personnel.

eAppendix 2. Reactogenicity Assessment

The occurrence of solicited reactogenicity events and unsolicited adverse events was assessed daily through post-vaccination Day 8 using a standard symptom diary. The severity grading is described in the Tables below.

Solicited Injection-site Reactogenicity

Symptom	Mild (Grade 1)	Moderate (Grade 2)	Severe (Grade 3)
**Pain**	Noticeable with no limitation in normal daily activity	Some limitation in normal daily activity	Completely unable to perform normal daily activity
**Tenderness**	Noticeable with no limitation in normal daily activity	Some limitation in normal daily activity	Completely unable to perform normal daily activity
**Shoulder Pain on Side of Vaccination**	Noticeable with no limitation in normal daily activity	Some limitation in normal daily activity	Completely unable to perform normal daily activity
**Induration/ Swelling**	Any to <25 mm	≥25 to <50 mm	≥50 mm
**Erythema/ Redness**	Any to <25 mm	≥25 to <50 mm	≥50 mm

Solicited Systemic Reactogenicity

Systemic	Mild (Grade 1)	Moderate (Grade 2)	Severe (Grade 3)
**Fever** (° C)<br>(° F)	≥37.5 - ≤38.0<br>≥99.5 - ≤100.4	>38.0 - ≤39.0<br>>100.4 - ≤102.2	>39.0<br>>102.2
**Chills**	Noticeable with no limitation in normal daily activity	Some limitation in normal daily activity	Completely unable to perform normal daily activity

© 2021 Schmader KE et al. *JAMA Network Open.*

**Systemic**	**Mild (Grade 1)**	**Moderate (Grade 2)**	**Severe (Grade 3)**
**Fatigue**	Noticeable with no limitation in normal daily activity	Some limitation in normal daily activity	Completely unable to perform normal daily activity
**Malaise**	Noticeable with no limitation in normal daily activity	Some limitation in normal daily activity	Completely unable to perform normal daily activity
**Myalgia**	Noticeable with no limitation in normal daily activity	Some limitation in normal daily activity	Completely unable to perform normal daily activity
**Arthralgia**	Noticeable with no limitation in normal daily activity	Some limitation in normal daily activity	Completely unable to perform normal daily activity
**Nausea**	Noticeable with no limitation in normal daily activity	Some limitation in normal daily activity	Completely unable to perform normal daily activity
**Vomiting**	Noticeable with no limitation in normal daily activity	Some limitation in normal daily activity	Completely unable to perform normal daily activity
**Diarrhea**	Noticeable with no limitation in normal daily activity	Some limitation in normal daily activity	Completely unable to perform normal daily activity
**Headache**	Noticeable with no limitation in normal daily activity	Some limitation in normal daily activity	Completely unable to perform normal daily activity

eTable. Local and Systemic Reactions during Days 1 through 8 Following Trivalent Adjuvanted Inactivated Influenza Vaccine (aIIV3) and Trivalent High-Dose Inactivated Influenza Vaccine (HD-IIV3) by Age Group in Older Adults.

Local Reactions	Ages 65-79 Years		Ages ≥80 Years	
	aIIV3 n=298 %	IIV3-HD n=294 %	aIIV3 n=80 %	IIV3-HD n=83 %
Injection-Site Pain				
Any	22.1	28.6	18.8	13.3
Moderate[a]	2.7	6.1	2.5	3.6
Severe[b]	0.7	0.3	0.0	0.0
Moderate-Severe	3.4	6.4	2.5	3.6
Tenderness				
Any	47.3	53.1	45.0	30.1
Moderate[a]	7.0	6.1	3.8	2.4
Severe[b]	1.0	0.3	0.0	0.0
Moderate-Severe	8.0	6.4	3.8	2.4

© 2021 Schmader KE et al. *JAMA Network Open.*

Swelling				
Any	11.4	13.9	11.3	9.6
Moderate[a]	1.3	5.8	2.5	3.6
Severe[b]	0.7	0.7	1.3	1.2
Moderate-Severe	2.0	6.5	3.8	4.8
Redness				
Any	8.1	9.2	7.5	10.8
Moderate[a]	0.7	2.4	2.5	2.4
Severe[b]	0.3	1.0	1.3	1.2
Moderate-Severe	1.0	3.4	3.8	3.6
Shoulder Pain Side of Vaccination				
Any	17.4	15.3	11.3	7.2
Moderate[a]	4.7	3.7	0.0	3.6
Severe[b]	0.7	0.3	1.3	0.0
Moderate-Severe	5.4	4.0	1.3	3.6

Systemic Reactions	Ages 65-79		Ages ≥80 Years	
	aIIV3 N=298 %	IIV3-HD N=294 %	aIIV3 N=80 %	IIV3-HD N=83 %
Fatigue				
Any	16.8	12.2	11.3	4.8
Moderate[a]	6.7	3.7	5.0	1.2
Severe[b]	1.0	1.0	0.0	0.0
Moderate-Severe	7.7	4.7	5.0	1.2
Malaise				
Any	12.8	10.2	10.0	3.6
Moderate[a]	3.7	2.4	5.0	0.0
Severe[b]	1.0	1.4	0.0	0.0
Moderate-Severe	4.7	3.8	5.0	3.6
Myalgia				
Any	12.1	11.2	10.0	7.2
Moderate[a]	4.7	4.4	2.5	2.4
Severe[b]	0.7	0.3	1.3	1.2

© 2021 Schmader KE et al. *JAMA Network Open.*

Moderate-Severe	5.4	4.7	3.8	3.6
Headache				
Any	13.1	12.6	5.0	2.4
Moderate[a]	1.3	2.0	0.0	0.0
Severe	1.0	0.7	0.0	0.0
Moderate-Severe	2.3	2.7	0.0	0.0
Arthralgia				
Any	10.7	9.5	7.5	3.6
Moderate[a]	4.0	2.4	2.5	1.2
Severe[b]	1.3	0.7	0.0	1.2
Moderate-Severe	5.3	3.1	2.5	3.4
Nausea				
Any	6.0	4.8	3.8	1.2
Moderate[a]	0.7	1.0	0.0	0.0
Severe[b]	0.0	0.7	0.0	0.0
Moderate-Severe	0.7	1.7	0.0	0.0
Vomiting				
Any	1.3	1.0	0.0	0.0
Moderate[a]	0.3	0.3	0.0	0.0
Severe[b]	0.3	0.3	0.0	0.0
Moderate-Severe	0.6	0.6	0.0	0.0
Diarrhea				
Any	6.0	4.8	2.5	4.8
Moderate[a]	1.3	2.0	0.0	2.4
Severe[b]	0.3	0.3	0.0	0.0
Moderate-Severe	1.3	2.3	0.0	2.4
Chills				
Any	5.0	4.1	1.3	2.4
Moderate[a]	1.0	1.7	0.0	0.0
Severe[b]	0.0	0.0	0.0	0.0
Moderate-Severe	1.0	1.7	0.0	0.0
Fever				
Any	3.4	4.5	2.5	2.4

© 2021 Schmader KE et al. *JAMA Network Open.*

Moderate[a]	0.0	1.0	0.0	0.0
Severe[b]	0.0	0.0	1.3	0.0
Moderate-Severe	0.0	1.0	1.3	0.0

[a]Moderate: Swelling and Redness: ≥25mm to <50mm; Fever >38 - ≤39°C

All other reactions: Some limitation in normal daily activity

[b]Severe: Swelling and redness: ≥50mm; fever >39°C

All other reactions: Completely unable to perform normal daily activity.

© 2021 Schmader KE et al. *JAMA Network Open.*
