# Supplementary material for: Safety, Reactogenicity, and Health-Related Quality of Life After Trivalent Adjuvanted vs Trivalent High-Dose Inactivated Influenza Vaccines in Older Adults: A Randomized Clinical Trial
Source: JAMA Netw Open. 2021 Jan 14;4(1):e2031266. doi: 10.1001/jamanetworkopen.2020.31266 (PMC7809592; doi:10.1001/jamanetworkopen.2020.31266)
Supplement: Supplement 3. — Data Sharing Statement [file jamanetwopen-e2031266-s003.pdf]

Data Sharing Statement

Schmader. Safety, Reactogenicity, and Health-Related Quality of Life After Trivalent Adjuvanted vs Trivalent High-Dose Inactivated Influenza Vaccines in Older Adults. *JAMA Netw Open*. Published January 14, 2021. doi:10.1001/jamanetworkopen.2020.31266

Data
Data available: No
